# Constraining modelled global vegetation dynamics and carbon turnover using multiple satellite observations

**DOI:** 10.1038/s41598-019-55187-7

**Published:** 2019-12-10

**Authors:** Matthias Forkel, Markus Drüke, Martin Thurner, Wouter Dorigo, Sibyll Schaphoff, Kirsten Thonicke, Werner von Bloh, Nuno Carvalhais

**Affiliations:** 1 Technische Universität Dresden, Institute of Photogrammetry and Remote Sensing, Helmholtzstr. 10, 01069 Dresden, Germany; 2 Potsdam Institute for Climate Impact Research, Telegraphenberg A 62, Potsdam, Germany; 3 Senckenberg Biodiversity and Climate Research Centre (SBiK-F), Senckenberg Gesellschaft für Naturforschung, Senckenberganlage 25, 60325 Frankfurt am Main, Germany; 4 TU Wien, Department of Geodesy and Geoinformation, Gusshausstr. 27-29, Vienna, Austria; 5 Max Planck Institute for Biogeochemistry, Hans-Knöll-Str. 10, Jena, Germany

**Keywords:** Carbon cycle, Biogeography, Ecological modelling

## Abstract

The response of land ecosystems to future climate change is among the largest unknowns in the global climate-carbon cycle feedback. This uncertainty originates from how dynamic global vegetation models (DGVMs) simulate climate impacts on changes in vegetation distribution, productivity, biomass allocation, and carbon turnover. The present-day availability of a multitude of satellite observations can potentially help to constrain DGVM simulations within model-data integration frameworks. Here, we use satellite-derived datasets of the fraction of absorbed photosynthetic active radiation (FAPAR), sun-induced fluorescence (SIF), above-ground biomass of trees (AGB), land cover, and burned area to constrain parameters for phenology, productivity, and vegetation dynamics in the LPJmL4 DGVM. Both the prior and the optimized model accurately reproduce present-day estimates of the land carbon cycle and of temporal dynamics in FAPAR, SIF and gross primary production. However, the optimized model reproduces better the observed spatial patterns of biomass, tree cover, and regional forest carbon turnover. Using a machine learning approach, we found that remaining errors in simulated forest carbon turnover can be explained with bioclimatic variables. This demonstrates the need to improve model formulations for climate effects on vegetation turnover and mortality despite the apparent successful constraint of simulated vegetation dynamics with multiple satellite observations.

## Introduction

Terrestrial ecosystems compensate currently for around 1/3 of all anthropogenic carbon emissions from fossil fuel burning, cement production and land use change^1^. However, it is uncertain if land ecosystems will remain a sink of carbon under future climate change conditions^2^. The uncertainty in the future land carbon uptake is related to how dynamic global vegetation models (DGVMs) account for net primary production (NPP), soil carbon decomposition, vegetation dynamics (i.e. processes that control changes in the area coverage of vegetation types), and vegetation carbon turnover^3^. While DGVMs generally predict an increase in NPP, the future changes in the terrestrial vegetation carbon storage, or biomass, differ largely among models^4^. These different future trajectories in simulated vegetation biomass and carbon turnover are related to various processes such as plant phenology, forest succession and regrowth, initiated by disturbances such as fires, and by drought and temperature effects on plant mortality^3–6^. For example, DGVMs do not sufficiently represent climate-induced effects on vegetation carbon turnover, e.g. through frost or drought stress and insect outbreaks, resulting in insufficiently modelled spatial patterns of vegetation biomass^6^. In contrast, regional patterns of forest biomass and turnover rates as derived from satellite-based products can largely be explained by climate variables and hence it should be feasible to accurately simulate these processes in DGVMs^7^.

Satellite observations provide information on several ecosystem properties that can potentially help to constrain model simulations of vegetation productivity, biomass and vegetation dynamics^8^. For example, decadal time series of vegetation greenness (i.e. normalized difference vegetation index, NDVI or the fraction of absorbed photosynthetic active radiation, FAPAR) are widely used to identify short- to long-term changes in land surface phenology and photosynthetic capacity^9–11^. Multi-year land cover maps provide information on spatial distributions and long-term changes of vegetation types^12,13^. Satellite retrievals of sun-induced fluorescence (SIF) are closely linked to gross primary production (GPP)^14,15^ and can constrain photosynthesis in DGVMs^16,17^. Satellite-derived maps of above-ground biomass provide information about the spatial distribution of vegetation carbon^18–20^. Biomass maps can be used together with data-based estimates of GPP and NPP to estimate total ecosystem and vegetation carbon turnover times, respectively^7,21,22^.

Previously, satellite observations of vegetation greenness or leaf area index have been intensively used to improve DGVM simulations of phenology and plant productivity^23–26^. Thereby optimization algorithms are used to estimate model parameters and their uncertainties within formal model-data integration approaches^27,28^ or more specifically within carbon cycle data assimilation systems^29–32^. For example, we previously optimized phenology, light absorption, and productivity-related parameters of the LPJmL (Lund-Potsdam-Jena managed Land) DGVM^33^ against 30 years of satellite-derived FAPAR, 10 years of vegetation albedo and a data-based estimate of mean annual GPP^23^. In addition to improvements of the model performance in comparison to these data sets, the optimization resulted also in a better representation of high-latitude tree cover and biomass^23^, and seasonal dynamics and trends in global productivity and atmospheric CO_2_ seasonality^34,35^. It has been also shown that the vegetation distribution in Russia as simulated by another variant of LPJ can be improved by optimizing parameters against a land cover map^36^. Hence, these studies suggest that it might be possible to jointly constrain the simulated vegetation productivity, dynamics and carbon turnover of a DGVM with satellite observations.

**Figure 1:**
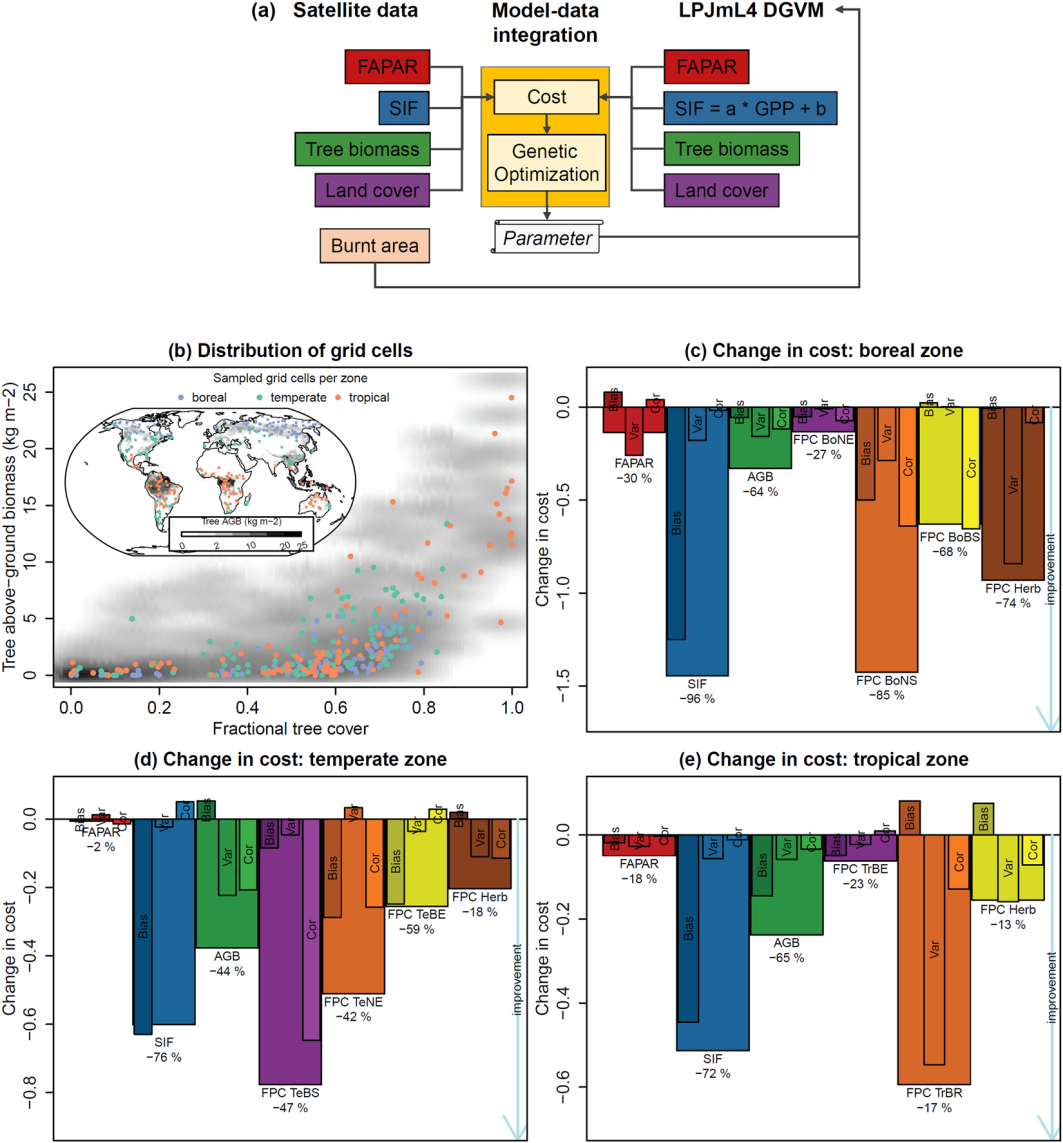
Overview of the model-data integration setup and changes in the model-data cost. Panel (a) shows how the used satellite data and the LPJmL model were integrated to estimate model parameters using a genetic optimization algorithm. Panel (b) shows the spatial distribution and the distribution with respect to tree above-ground biomass and fractional tree cover of grid cells that were used for the three optimization experiments in the boreal, temperate and tropical zones. Panels (c–e) show for each optimization the changes in the cost for each data set and for the components of the cost that are related to model-data bias, variance ratio and correlation.

Here, we aim to explore how the combined information from satellite data on FAPAR, SIF, above-ground biomass of trees, and tree cover distribution can be used to constrain parameters of the LPJmL (version 4.0)^33^ DGVM and hence to improve simulations of regional to global vegetation distribution and carbon turnover (Fig. 1a). Based on these satellite datasets, we compute a multivariate cost function (i.e. model-data error, see Methods) to optimize model parameters that regulate the simulated phenology, photosynthesis, vegetation carbon turnover, establishment, mortality and bioclimatic limits of plant functional types (PFTs) (see Supplementary Table S1). In addition, we directly prescribe a satellite data set of burned area into the fire module of LPJmL4 to constrain the occurrence and spatial extent of fires with observations. Our approach could provide the basis for new state-of-the art strategies to improve parameterizations of and simulations from DGVMs.

## Results

### Effects of individual data sets on changes in the multivariate model-data cost

We performed three independent optimization experiments for the boreal, temperate and tropical zones, respectively (Fig. 1b). The optimization resulted in all zones and for all data sets in a reduced cost, i.e. an improved model performance (Fig. 1c–e). For all climatic zones, the cost reductions were largest for SIF, PFT fractions and biomass. The smallest reductions occurred for FAPAR, against which LPJmL had been optimized previously^23^. The used cost function allows quantifying the contribution of changes in model-data bias, variance ratio, and correlation on the overall cost. For example, the cost for SIF was in all zones reduced mostly because of a reduction in the bias whereas variance and correlation had only small changes. The changes in the cost of biomass and PFT fractions were caused by regionally diverse changes in bias, variance ratio or correlation.

In the boreal zone, the reductions in cost were largest for SIF and for the coverage of the summer-green tree and herbaceous PFTs (Fig. 1c). For FAPAR, the cost was reduced because of an improved variance but an increase in bias and a decline in correlation occurred.

In the temperate zone, reductions in the cost were largest for the coverage of broad-leaved summer-green and needle-leaved evergreen tree PFTs, SIF and biomass (Fig. 1d). Despite the overall improvement for the biomass, there was an increased bias. As biomass was included as a static map in the optimization, this result points towards that biomass will be under- or overestimated in the temperate zone although the spatial distribution (indicated by improved correlation) and variability (indicated by improved variance) were better reproduced.

In the tropical zone, the cost reduced mostly because of an improved variance of the rain-green tree PFT (Fig. 1e). However, we found that the optimum parameter set and other individual parameter sets with low total costs generally coincided with a bias in herbaceous vegetation cover and an increased error for broad-leaved evergreen tree cover (Supplementary Fig. S1). When we used the optimum parameter set in a global model run, we found that across the tropical Savannah regions, tree cover was generally over- and herbaceous cover underestimated. Therefore, we selected an alternative individual parameter set from the optimization results with a bias component <0.1 for herbaceous vegetation cover and with reduced costs for the tree PFTs, for the bias in biomass and for the correlation in SIF and FAPAR (Supplementary Fig. S1a). Unlike the initial “optimal” parameter set, the selected parameter set resulted in reduced costs for all data sets and had even slightly better performances for biomass and broad-leaved evergreen tree cover (Fig. 1e shows the results for the selected parameter set). We then used the best-performing parameter sets (i.e. the optimum for the temperate and boreal zone and the selected set for the tropical zone, Supplementary Table S2) to run and evaluate a global model simulation.

### Data constraints on parameter values and uncertainties

**Figure 2:**
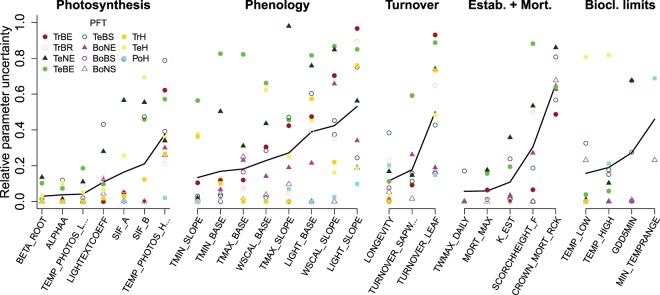
Relative uncertainty of model parameters after optimization, grouped by processes. The relative uncertainty is the ratio of posterior/prior uncertainty. Low and high values of relative uncertainty indicate strongly and weakly constrained parameters, respectively. All parameters are defined by PFT (coloured dots). The black lines are added to support visual interpretation and show the relative uncertainty of each parameter averaged across PFTs. Note that for the tropical PFTs the uncertainties refer to the optimized parameter set but are not associated to the selected best parameter set that is used for global model simulations.

Generally, we found various PFT-dependent changes in model parameter values (Fig. 2, Supplementary Figs. S2–3). By investigating the uncertainty of model parameters after optimization, we are able to identify which parameters were well constrained with the used model-data integration framework. In the following, we use the term “well-constrained” for parameters that had a posterior uncertainty of <20% relative to the prior uncertainty (Fig. 2, see Eq. 3 in Methods). Broadly speaking, photosynthesis-related parameters were better constrained than parameters that control phenology, turnover, establishment, mortality and bioclimatic limits. Among the photosynthesis-related parameters, parameters that control the SIF-GPP relationship were well constrained for tropical and boreal PFTs but had larger uncertainties for temperate PFTs. The upper limit for the temperature optimum of photosynthesis was generally weakly constrained.

In addition, some parameters that control phenology, turnover, mortality or bioclimatic limits were well constrained. Phenology-related parameters control the sensitivity of leaf development and senescence to temperature, water availability or light. The temperature-related parameters were best constrained. Water-related and light-related phenology parameters were well constrained for PFTs that grow in water- (tropical herbaceous and rain-green tree PFTs, boreal summer-green PFT) and light-limited (boreal PFTs) climates, respectively. Phenology parameters were generally poorly constrained for the temperate evergreen tree PFT.

Leaf longevity, the turnover time of sapwood to heartwood, and most establishment and mortality-related parameters were well constrained. Parameters that control fire-induced mortality were poorly constrained. Bioclimatic limits, especially the lower and upper temperature limits for establishment and survival of PFTs, were well constrained for most boreal and temperate PFTs.

We then investigated which parameters were causing the increasing bias in herbaceous vegetation cover in the tropical optimization experiment. We found that the bias in herbaceous cover was especially related to a parameter that controls the phenology of the tropical herbaceous PFT at high temperatures (i.e. TMAX_BASE_TrH) and to the leaf longevity and light extinction coefficient parameters of TrH as well as one parameter that controls the SIF-GPP relationship in the broadleaved rain-green PFT (Supplementary Fig. S4). All of these parameters mostly affect the magnitude and dynamics of FAPAR and hence of GPP and SIF. Overall, our results demonstrate that parameters for productivity, phenology and vegetation dynamics within a DGVM can be jointly estimated from the multitude of satellite observations.

### Improved simulations of global vegetation distribution and biomass

**Figure 3:**
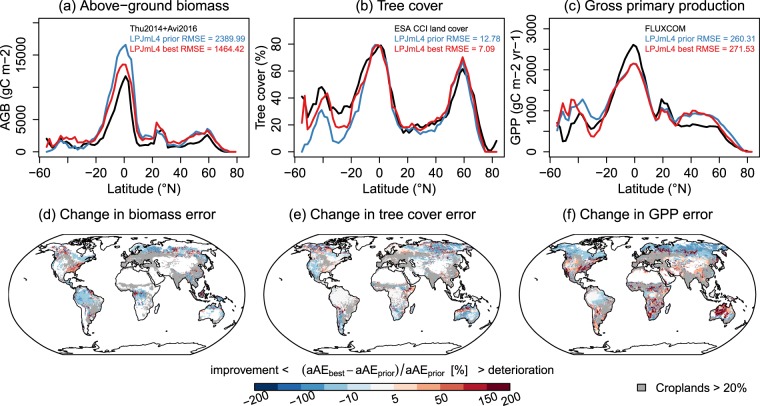
Global patterns of mean annual aboveground biomass (2009–2011), tree cover (1992–2015) and gross primary production (1982–2010). Panels (a–c) show latitudinal gradients from the LPJmL prior and best model runs and from each reference dataset. Panels (d–f) show percentage changes in the absolute average error ($aAE=|\bar{s}-\bar{o}|$) whereby blue colours indicate that LPJmL improved after the optimization. Regions with >20% cropland cover are masked (grey colours).

The optimized model better reproduced the global distribution of above-ground tree biomass and tree cover, and of GPP in northern latitudes (Fig. 3). While LPJmL with original parameters (LPJmL-prior) overestimated biomass globally, the optimized model had a better performance especially across the tropical and boreal forests (Fig. 3a,d). However, the optimized model had a deteriorated performance for biomass in some regions, especially in the eastern United States. Globally, the optimized runs had improved description of total tree cover, especially across the arctic-boreal regions and in temperate and tropical semi-arid regions (Fig. 3b,e). The prior model overestimated GPP in comparison to the independent FLUXCOM^37^ product in temperate and boreal regions. After the optimization, this overestimation was substantially reduced across the boreal zone and in some parts of tropical forests (Fig. 3c,f).

The simulated spatial distribution of PFTs in the optimized model was more similar to the observations than the prior model across large areas (Supplementary Fig. 5). The simulated PFT distribution especially improved in large parts of Siberia, Africa and the Amazon. The optimized model reproduced better the coverage of broad-leaved evergreen trees in the tropics, of broad-leaved deciduous trees in northern high latitudes (>50°N) and of needle-leaved evergreen trees in northern mid-latitudes (20–40°N) (Supplementary Fig. 6). However, the optimization resulted in an overestimation of the coverage of needle-leaved deciduous trees in boreal forests of North America and in an underestimation of herbaceous cover in Australia and parts of southern Africa **(**Supplementary Figs. 6 and 7**)**.

Simulated FAPAR, SIF and GPP from both the prior and the optimized model were highly correlated (r > 0.8) with observations across many regions (Supplementary Fig. 8). Weak correlations (r < 0.3) occurred for all three variables over tropical forests where optical satellite observations are generally hampered by cloud cover. Interestingly, the correlations for SIF and GPP were higher than for FAPAR which shows that LPJmL can better capture dynamics in productivity than in seasonal canopy development. The correlation with SIF and GPP did not change between the prior and optimized model in many regions but the optimized model had weaker correlations in various arid regions. The correlations with FAPAR improved in the optimized model in boreal forests but deteriorated in arctic and semi-arid regions.

### Impacts on simulated global carbon cycling and vegetation carbon turnover

**Figure 4:**
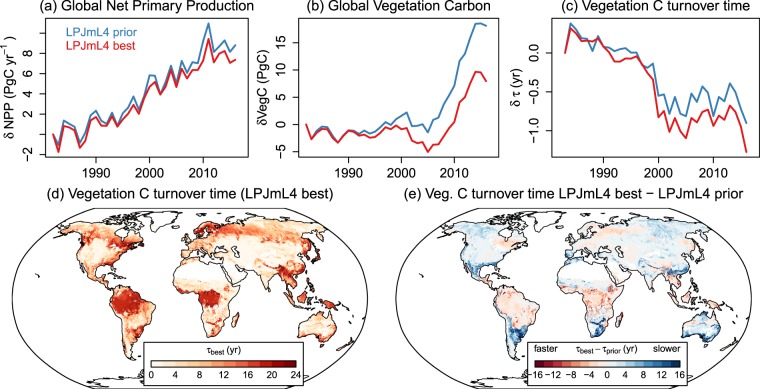
Effect of the model optimization on global vegetation carbon turnover. Shown are global annual totals of (**a**) net primary production, (**b**) vegetation carbon and (**c**) vegetation carbon turnover times for the LPJmL4 prior and best models runs relative to the values in 1982. Panel (d) shows global pattern of vegetation carbon turnover time from the LPJmL4 best model run, calculated by assuming the steady-state assumption. Panel (e) shows the change in vegetation carbon turnover time between the LPJmL best and prior model runs.

Globally, the optimized model simulated lower carbon fluxes and stocks than the prior model (Table 1). For example, global GPP was reduced by 6%, NPP by 9% and vegetation carbon stocks by 7% and remains within the uncertainty range of global data-driven estimates^38^. However, both versions of LPJmL simulated higher fire carbon emissions (+86% and +59% for the prior and optimized model) than the estimates from the Global Fire Emissions Database^39^. Note that the simulated fire carbon emissions are not confounded by potential limitations of the model to simulate the occurrence and extent of fires because we prescribed observed burned area to both model simulations. The prior model had clearly higher global vegetation carbon stocks (543.5 PgC) than suggested by the uncertainty limits (343–539 PgC) of a global satellite-derived estimate^21^. The optimized model (504.6 PgC) was within this uncertainty range. Despite these changes in total carbon stocks and fluxes, the optimization affected also the magnitude of carbon cycle trends: The optimized model had weaker positive trends in global NPP and biomass but stronger negative trends in vegetation carbon turnover time than the prior model (Fig. 4a–c).

**Table 1:** Global carbon fluxes, stocks and turnover times as simulated by LPJmL averaged for the period 1982–2016.

	GPP (PgC yr^−1^)	NPP (PgC yr^−1^)	FireC (PgC yr^−1^)	Rh (PgC yr^−1^)	VegC (PgC)	τ_veg_ (yr)	SoilC (PgC)
LPJmL prior	125.3	56.4	4.1	43.5	543.5	9.6	1898
LPJmL best	117.4	51.3	3.5	39.1	504.6	9.9	1456
Reference	123 (102–135)^A^^38^		2.2 (1.8–3)^B^^39^		442 (343–539)^A^^21^		2397 (1837–3257)^A^^21^

GPP: gross primary production, NPP: net primary production, FireC: fire carbon emissions, Rh: heterotrophic respiration, VegC: vegetation carbon, τ_veg_; vegetation carbon turnover time, SoilC: soil carbon.

^A^Ranges are 95% confidence intervals as reported in the references; ^B^The range is the reported annual minimum (2013) and maximum (1997) value^39^.

**Figure 5:**
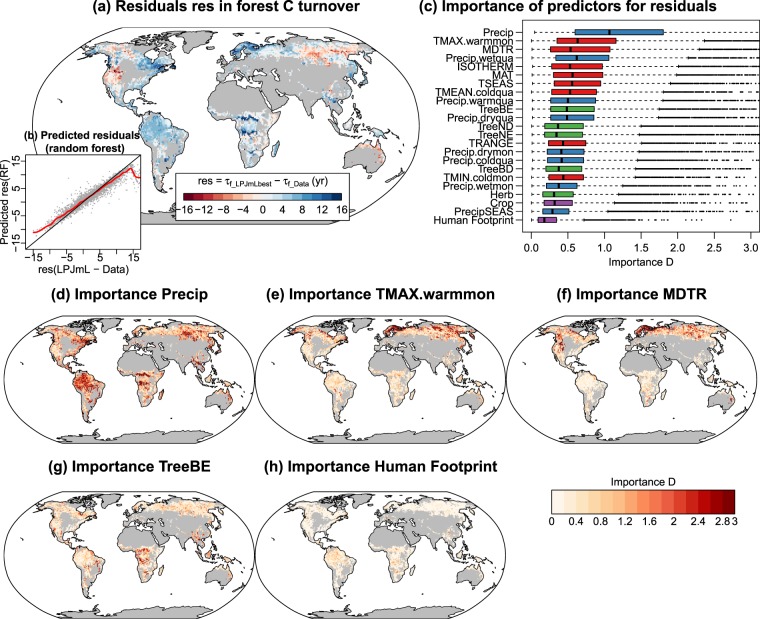
Explanation of residuals in simulated forest carbon turnover time (τ_f_) from the optimized LPJmL model using the random forest machine learning approach. (**a**) Spatial distribution of τ_f_ residuals between simulations from the LPJmL-best model run and from data-derived estimates. Grey areas are croplands (>20%) or non-forests (tree cover < 40%). (**b**) The residuals in τ_f_ between LPJmL and data-derived estimates were re-constructed from several predictor variables (bioclimatic variables, observed land cover and the human footprint index) using the random forest (RF) machine learning algorithm (MEF = 0.96). (**c**) Distributions of the grid cell-level importance of predictor variables in RF. Variable importance D is defined as the RMSE per grid cell between RF predictions and RF predictions after perturbing the selected predictor variable. Annual precipitation has the highest importance in predicting model-data residuals (**d**). As further examples, are shown the importance of maximum temperature in the warmest month (**e**), mean monthly diurnal temperature range (**f**), broad-leaved evergreen tree cover (**g**), and the human footprint index (**h**).

The optimized model had globally a slightly higher vegetation carbon turnover time (+3%) than the prior model because of the slightly stronger relative changes in global vegetation carbon stocks than in productivity. Globally, the changes in vegetation carbon turnover time were clearly dominated by changes in biomass. However, the changes in vegetation turnover time between the prior and the optimized model varied regionally: increased turnover time in boreal forests and in some semiarid regions and decreased turnover time in tropical regions (Fig. 4d,e). In order to evaluate the simulated vegetation carbon turnover in the optimized model, we computed an approximation of forest carbon turnover time (τ_f_) from simulations and from the AGB and GPP datasets (see Methods). LPJmL overestimated τ_f_ in most tropical forests and in parts of boreal forests but underestimates τ_f_ in central and eastern Siberia, in western North America and northern Australia (Fig. 5a). We then applied the random forest (RF) machine learning approach to explain the residuals (LPJmL – data) in τ_f_ with bioclimatic, land cover, and human-related predictor variables. RF is able to reconstruct the model-data residuals mainly by using climate-related predictors (MEF = 0.96, Fig. 5b,c). Globally, annual precipitation is the most important predictor for τ_f_ residuals and is most important in tropical forests and in southern boreal forests (Fig. 5d). Given the overestimation of biomass in tropical forests, this indicates that LPJmL has a too high sensitivity of above-ground biomass and hence of forest carbon turnover to precipitation. The overestimation of τ_f_ in some parts of boreal forests is strongly related to the maximum temperature of the warmest month and to diurnal temperature range, indicating that the model approach in LPJmL underestimates the role of heat and drought effects on forest carbon turnover (Fig. 5e,f). Land cover- and human related variables were of minor or of only local importance (Fig. 5g,h).

We previously showed that most DGVMs (including a previous version of LPJmL) poorly represent relations between forest carbon turnover rates and climate variables in temperate and boreal forests^6,7^. We here repeated these analyses with results from the prior and optimized LPJmL model (Supplementary Information 3). The new results showed that relations between forest turnover rate and the number of icing days and the maximum length of warm-dry periods did not change between the prior and optimized model (Supplementary Figs. 10 and 11). Overall, our results demonstrate that errors in simulated vegetation carbon turnover after model optimization can be predicted from climate data. This indicates that LPJmL misses regionally important disturbances and plant-stress functions such as frost damage, drought and heat effects on mortality in boreal and tropical forests. Hence, the simulated vegetation dynamics and carbon turnover in LPJmL cannot be improved further with parameter estimation but requires the improvement of model structures.

## Discussion

In summary, the use of satellite-derived datasets of FAPAR, SIF, above-ground biomass, land cover, and burned area within a joint model-integration framework constrained simulated global and regional patterns of GPP, biomass and tree cover in LPJmL. The optimization most strongly constrained model parameters for photosynthesis and some parameters controlling phenology, turnover, mortality and bioclimatic limits. Hence, our results demonstrate the feasibility of constraining key aspects of vegetation dynamics in a DGVM with satellite observations and helps to identify missing process representations.

In agreement with MacBean *et al*.^16^, we found that SIF data can strongly constrain simulated GPP in a DGVM. As we were using datasets that are representative for both photosynthetic carbon uptake (FAPAR and SIF) and vegetation carbon turnover (AGB, land cover and burned area), we were also able to constrain many model parameters that control carbon stocks and different processes of carbon turnover such as phenology, mortality and bioclimatic limits. Novel datasets on leaf and sapwood biomass^40^ could further help to constrain parameters that control different biomass compartments. However, the largest uncertainty in the size of land carbon stocks is largely caused by different data-based estimates for soil carbon^21,41^. Given these uncertainties, we did not include estimates of soil carbon in the model-data integration to potentially constrain total-ecosystem carbon turnover. However, unlike the recent study by Wu *et al*.^41^, we demonstrate that land carbon cycle simulations in DGVMs can be improved with state-of-the art datasets on biomass. In agreement with Wu *et al*., we found the largest improvements for biomass in northern ecosystems. Contrary to our results, Wu *et al*. report that simulated biomass can be only weakly constrained in tropical forests within their model-data integration framework because of large differences between the two used AGB datasets. We here rely only on one dataset of tropical AGB, which shows better agreement with reference data than other data sets^18^. Having said that, it is currently not possible to use realistic uncertainty estimates of large-scale tropical biomass in model-data integration because all available data sets are not independent of each other.

Our results reveal diverse changes in simulated vegetation distribution and carbon turnover. A challenging result of the optimization is the overestimation of tree cover and the corresponding underestimation of herbaceous cover in savannah regions. The observed bimodal distribution of tree cover in savannah regions has been previously explained through fire feedbacks^42^. Here we prescribed observed burned area to LPJmL to represent fire feedbacks on vegetation realistically. However, globally consistent time series of burned area are only available since 2000. As we recycled the observed burned area from the years 2000–2016 before 2000, we might overestimate real fire return intervals but might underestimate total burned area before 2000 given that burned area declined in Savannah regions like the Sahel^43^. Altogether, this might result in a misrepresentation of post-fire regrowth trends. However, given the relatively short fire return interval in savannahs, our results suggest moreover that fire impacts on vegetation (i.e. combustion, post-fire mortality) or the adaptation of vegetation to fires are not sufficiently represented to yield an accurate distribution of woody and herbaceous vegetation in savannahs. A similar result was recently found for the JSBACH model^44^. Another reason for the overestimation of tree cover might be the abundance of animals that strongly regulate the productivity and biomass in savannah ecosystems^45^, which is however not considered in most DGVMs. Hence, the inappropriate simulation of tree and grass cover distributions suggests that the effect of abiotic and biotic disturbances on ecosystem carbon stocks need to be revised in DGVMs to accurately simulate savannah ecosystems.

We found that model-data errors in forest carbon turnover after the optimization of LPJmL are still related to bioclimatic variables. The importance of precipitation and of maximum temperatures in explaining these errors suggest that drought- and heat effects on turnover and mortality need to be improved as already suggested by several previous studies^6,46,47^. Currently, bioclimatic limits are used in DGVMs to allow the establishment and mortality of PFTs and hence to control the spatial distribution of vegetation types. From a process-oriented point, bioclimatic limits prescribe the effect of biotic and abiotic disturbances or vegetation sensitivity to frost or heat on mortality in a simple manner. The random forest-based results allow identifying regional drivers of forest carbon turnover and hence suggest that the current use of bioclimatic limits in DGVMs should be rather replaced with more mechanistic functions that relate carbon turnover or mortality to extreme climate conditions.

Furthermore, biotic disturbances and forest management are equally important for tree mortality like fire but are not considered in LPJmL (and in most other DGVMs)^48^. Historic mortality events and past forest management cause a regrowth of forests and are important for the current land carbon sink^5^. Hence, an accurate simulation of regrowth and carbon uptake trends requires that models can sufficiently simulate mortality in response to climate variability and that management-related vegetation changes can be prescribed from data sets. Current-generation global multi-temporal satellite-based land cover data sets realistically depict regional trends in forest cover^12,13^. However, it is unclear if such datasets contain signals of forest mortality. Regionally, the use of higher resolution satellite imagery allows mapping forest disturbances over decades^49^ but such datasets are not readily available over large scales for use in DGVMs. Recent developments to map biomass and forest structure will help to better constrain dynamics and changes in forest carbon turnover^50,51^.

In conclusion, we show that simulated global vegetation dynamics and turnover from current-generation DGVMs can be constrained through a joint use of satellite observation of vegetation greenness, sun-induced fluorescence, biomass, land cover and burned area. To exploit the full potential of model-data integration approaches in the future, better quantifications of data set uncertainties, novel large-scale and long-term datasets on ecosystem disturbances, and improved representations of mortality processes in DGVMs are needed. Thereby, machine-learning approaches can help to identify model deficiencies^52^ and to potentially derive improved model formulations for climate effects on vegetation carbon turnover.

## Methods

### LPJmL dynamic global vegetation model

LPJmL is a process-oriented DGVM that simulates global vegetation distribution, carbon and water fluxes and stocks in natural and agricultural land ecosystems^33^. Here we use version 4 of LPJmL^33^ which is based on the original LPJ model^53^ and its extension for agricultural areas^54^, fire^55^, permafrost^56^, and phenology^23^. LPJmL simulates the land coverage by different plant functional types (PFT) as the so called foliar projective cover (FPC)^53^. Changes in the FPC depend on changes in biomass, and on the establishment or mortality of individuals. Establishment and mortality depend on bioclimatic limits that control the ability of a PFT to grow or to survive under specific climatic conditions^53^. Additionally, mortality can occur because of heat stress, low productivity, fire, and age (expressed as background mortality). If mortality occurs, a certain number of individuals is killed, FPC reduced and the corresponding biomass is transferred to the litter carbon pools. FPC, FAPAR, GPP, and biomass form a positive vegetation cover-productivity-biomass feedback in LPJmL that is modified through changes in phenology, establishment, and mortality.

In order to use SIF observations in the model optimization, we additionally need to compute SIF in LPJmL. Based on previous work that used linear relationships between SIF and GPP for model optimization^16^, we compute SIF as:

1 $$SIF=a\ast GPP+b$$

The regression slope a and intercept b were treated as model parameters in LPJmL. A list of all model parameters that was included in this study is given in Supplementary Table S1.

LPJmL was run at 0.5° spatial resolution by using the default input data and spin up procedures^33,35^. Different to the default setup, we here used daily climate input data from the GLDAS reanalysis dataset (version 2.0 for the period 1948–2000 and version 2.1 between 2000 and 2016)^57,58^. For the model spinup we recycled the climate data for the period 1948 to 1977. The spinup was first computed for 5000 years by only simulating natural vegetation. Then we restarted the model from these conditions and computed a second spinup for 390 years with historical land use change data. The transient model run was then restarted from the conditions after the second spinup and simulated for the period 1948 to 2016. During the optimization, we re-started all iterations from the first spinup but repeated each time the second spinup. This is sufficient to bring vegetation carbon pools into a new equilibrium caused by the new model parameters.

### Satellite data sets

We used satellite datasets on FAPAR, SIF, PFT cover and aboveground biomass to optimize LPJmL (Supplementary Table S3). FAPAR was taken from the Moderate-resolution Imaging Spectroradiometer (MODIS) MOD15A2 product^59^. FAPAR observations for the period 2000 to 2015 were averaged to monthly time steps and to 0.5° resolution.

SIF data was taken from the GlobFluo product based on measurements from the Global Ozone Monitoring Experiment-2 (GOME-2) instrument. The retrieval algorithm is described by Köhler *et al*.^60^. Monthly SIF data was used for the period 2007 to 2014.

Estimates of above-ground forest biomass (AGB) were taken from forest biomass maps for the tropics^18^ and for northern forests^19^. The tropical biomass map is approximately representative for the period 2000–2010 and the northern biomass map is based on measurements from the Advanced Synthetic Aperture Radar (ASAR) instrument on board the Envisat satellite between October 2009 and February 2011. To compare these forest biomass maps with LPJmL, we computed the average above-ground biomass of tree PFTs for the years 2009 to 2011 from LPJmL simulations.

The coverage of PFTs was derived from the PFT maps based on the European Space Agency (ESA) Climate Change Initiative (CCI) land cover dataset (ESA Land cover_cci v 2.0.7)^12^. These PFT maps include the fractional cover of trees, shrubs, natural grass and crops and separate tree types by leaf longevity (i.e. evergreen vs. deciduous) and leaf type (broadleaf vs. needleleaf). We added the cover of shrubs to the corresponding tree PFTs because LPJmL does not separate between these growth forms. As LPJmL separates PFTs by climate zone (i.e. boreal, temperate and tropical PFTs), we reclassified the PFT maps to the PFT nomenclature of LPJmL using the Köppen-Geiger classification^61^ (Supplementary Table S4 and Fig. S9).

We used a satellite dataset of burned area to directly prescribe the occurrence, timing and spatial extent of fires in the LPJmL-SPITFIRE fire module^55^. Burned area was taken from the ESA CCI fire datasets which is based on MODIS observations for 2001 to 2016 (ESA Fire_cci v 50)^62^.

An independent data-driven estimate of gross primary production (GPP) was used to evaluate global model simulation (FLUXCOM Meteo dataset, 1982–2010)^37^. All satellite datasets were aggregated to 0.5° × 0.5 resolution of LPJmL.

### Model-data integration setup

To optimize LPJmL model parameters, we combined satellite observations and LPJmL simulations in a multi-variable cost function (Fig. 1a). The cost function includes time series of monthly FAPAR (2000–2015) and SIF (2007–2014), annual time series of FPC per tree PFT (1992–2015), mean annual FPC of all herbaceous PFTs (averaged over 1992–2015), and mean annual biomass (averaged over 2009–2011). As cost function, we used a modification of the Kling-Gupta efficiency (KGE)^63^. KGE is based on the Euclidean distance in a three-dimensional space of model performance measures that account for the bias, ratio of variance and correlation between simulations *s* and the observations *o*. We extended the KGE by defining it for multiple data sets *d*:

2 $$Cost=\sqrt{\mathop{\sum }\limits_{d=1}^{N}({(\frac{\overline{{s}_{d}}}{\overline{{o}_{d}}}-1)}^{2}+{(\frac{{\sigma }_{\{s,d\}}}{{\sigma }_{\{o,d\}}}-1)}^{2}+{(r({s}_{d},{o}_{d})-1)}^{2})}$$

where $\bar{s}$ and $\bar{o}$ are mean values (bias component) over space (i.e. different grid cells) and time (e.g. months) of simulations *s* and the observations *o*, respectively. *σ*_*s*_ and *σ*_*o*_ are variances (variance component) and *r* is the Pearson correlation coefficient over space and time. To account for the spatial-temporal data uncertainty in the cost function, we computed the weighted mean, weighted variance and weighted correlation by using the uncertainty of each observation as weights (w = 1/unc). This implies that uncertainties are considered only for observations within a dataset but potential differences in the uncertainty between data sets are not considered. We belief that this in an appropriate choice because uncertainty estimates from different data sets (and hence different uncertainty estimation approaches) are not comparable.

We used a genetic optimization algorithm (GENOUD^64^) to estimate model parameters. A more detailed description of the application of this algorithm for LPJmL can be found in our previous work^23^. For each zone (tropical, temperate or boreal), we ran the optimization algorithm for approximately 25 generations and 1000 individual parameter sets per generation. We included several LPJmL parameters in the optimization that regulate leaf phenology, light absorption, photosynthesis, biomass turnover, background mortality, heat stress mortality, mortality from fire, and bioclimatic limits for the establishment and survival of PFTs (Supplementary Table S1). All parameters are defined per PFT. The prior values for all parameters are taken from Schaphoff *et al*.^33^ and uniform prior uncertainties are defined by lower and upper boundaries of each parameter (Supplementary Figs. S2–3). The relative uncertainty *U* of a parameter after optimization as shown in Fig. 2 was computed as:

3 $$U=\frac{({u}_{o}-{l}_{o})}{({u}_{p}-{l}_{p})}$$

where u_p_ and l_p_ are the upper and lower boundaries of the prior parameter range and u_o_ and l_o_ are the maximum and minimum values of the parameter from the individual parameter sets with low cost that emerged during the optimization (i.e. cost < percentile 5% of the costs from all individual parameter sets).

Burned area was directly prescribed to the LPJmL-SPITFIRE fire module, i.e. the simulated burned area was replaced with observations and only fire intensity, fuel consumption and fire emissions were simulated.

### Optimization experiments and spatial sampling

We performed a multi-site optimization of LPJmL, i.e. model parameters were estimated by running the model and computing the cost function based on multiple grid cells at once. Unlike in previous studies that did multi-site optimizations for grid cells where one PFT dominates^23,26,65^, we here selected grid cells in which several PFTs co-occur to account for potential competition between PFTs and to constrain vegetation dynamics. However an optimization of all PFTs at once increases the number of target parameters and hence might hamper the possibility to estimate parameters. As a trade-off, we performed three optimization experiments for the PFTs of the boreal, temperate, and tropical zones, respectively:

- Boreal zone with the following PFTs: boreal needle-leaved evergreen trees (BoNE), boreal needle-leaved summer-green trees (BoNS), boreal broad-leaved summer-green trees (BoBS) and polar herbaceous vegetation (polar C3 grasses, PoH);
- Temperate zone: temperate needle-leaved evergreen trees (TeNE), temperate broad-leaved summer-green trees (TeBS), temperate broad-leaved evergreen tree (TeBE) and temperate herbaceous vegetation (temperate C3 grasses, TeH);
- Tropical zone: tropical broad-leaved evergreen trees (TrBE), tropical broad-leaved rain-green PFTs (TrBR) and tropical herbaceous vegetation (C4 grasses, TrH).

We sampled 102 grid cell per zone (Fig. 1b). The sampling was done randomly but stratified by PFTs, by the statistical distribution of above-ground biomass per PFT, and by grid cells that are likely representative for vegetation dynamics (i.e. grid cell with multiple PFTs, without a dominant PFT, or with large fires) (Supplementary Information SI5). The sampled grid cells cover the global and zonal distributions of tree above-ground biomass and tree cover (Fig. 1b). After the three optimization experiments, we used the best-performing parameter sets from each zone to make a global model run and to evaluate model results for all global grid cells (excluding agricultural areas, i.e. cropland cover >20%) (Supplementary Information SI2).

### Analysis of carbon turnover times and residuals

To analyse the effect of the model optimization on simulated carbon turnover, we computed vegetation carbon turnover time τ_Veg_ assuming the steady state assumption^21^:

4 $${\tau }_{Veg}=\frac{{\rm{VegC}}}{{\rm{NPP}}}$$

where VegC is the total carbon in vegetation (above- and belowground) and NPP is the net primary production. This definition is used for Table 1 and for the maps in Fig. 4. Vegetation turnover time as shown in Fig. 4c were calculated as following^4^:

5 $${\tau }_{Veg}=\frac{VegC}{\Delta VegC-NPP}$$

where ΔVegC is the difference in total vegetation carbon between consecutive years.

We also evaluated residuals between vegetation carbon turnover times from the optimized model and from data-derived estimates (Fig. 5a). As the satellite-derived biomass map for the tropics only represents above-ground biomass of trees^18^ and as we do not have a data-derived estimate on above-ground NPP, we performed this evaluation only for forested grid cells (tree cover > 40%) and approximated the forest turnover time as ${\tau }_{f}=AG{B}_{tree}/(GPP\times 0.5)$ using the FLUXCOM GPP dataset. A more accurate analysis including above- and belowground biomass and NPP has been done previously using the biomass map for temperate and boreal forests, which includes estimates of below-ground root biomass^6^. We here also repeated this analysis for the temperate and boreal forests with the simulations from the LPJmL-prior and -best runs (Supplementary Information SI3).

We then tested if the residuals in τ_f_ from the optimized LPJmL model can be explained to identify potential model limitations. Therefore we applied the random forest machine learning approach^66^ to predict model-data residuals from a suite of bioclimatic variables^67^, land cover (i.e. the observation-based PFT maps), and the human footprint index for the year 2009, which describes the human pressure on the environment^68^. We then computed the grid cell-level importance of each predictor variable for the performance of the RF using a recently developed approach^52^. Here the importance D of a predictor variable is computed for each grid cell from the original RF-predictions of the LPJmL vs. data residuals (res_0_) and from the RF-predictions of the LPJmL vs. data residuals after perturbing a predictor variable (res_p_):

6 $$D=\sqrt{{(re{s}_{p}-re{s}_{0})}^{2}}$$

## Supplementary information

Supplementary Information

## Data Availability

The used satellite datasets are available from the references listed in Supplementary Table 3. LPJmL4 model code and the used model-data integration package is available from https://github.com/PIK-LPJmL/LPJmL and https://github.com/PIK-LPJmL/LPJmLmdi, respectively.
