# Supplementary material for: Constraining modelled global vegetation dynamics and carbon turnover using multiple satellite observations
Source: Sci Rep. 2019 Dec 10;9:18757. doi: 10.1038/s41598-019-55187-7 (PMC6904745; doi:10.1038/s41598-019-55187-7)
Supplement: Supplementary file 1 — Supplementary Information [file 41598_2019_55187_MOESM1_ESM.docx]

Constraining modelled global vegetation dynamics and carbon turnover using multiple satellite observations

Supplementary Information

Matthias Forkel ^1,*^, Markus Drüke ^2^, Martin Thurner ^3^, Wouter Dorigo ^4^, Sibyll Schaphoff ^2^, Kirsten Thonicke ^2^, Werner von Bloh ^2^ and Nuno Carvalhais ^5^

^1^ Technische Universität Dresden, Institute of Photogrammetry and Remote Sensing, Helmholtzstr. 10, 01069 Dresden, Germany

^2^ Potsdam Institute for Climate Impact Research, Telegraphenberg A 62, Potsdam, Germany

^3^ Senckenberg Biodiversity and Climate Research Centre, Senckenberganlage 25, Frankfurt am Main, Germany

^4^ TU Wien, Department of Geodesy and Geoinformation, Gusshausstr. 27-29, Vienna, Austria

^5^ Max Planck Institute for Biogeochemistry, Hans-Knöll-Str. 10, Jena, Germany

***** Correspondence: matthias.forkel@tu-dresden.de; Tel.: +49 351 463-32270

Contents

[Figures 1](#_Toc4761207)

[Tables 2](#_Toc4761208)

[SI 1: LPJmL model parameters and optimization results 3](#_Toc4761209)

[SI 2: Global model evaluation 9](#_Toc4761210)

[SI 3: Regional relations between climate and forest C turnover rates 12](#_Toc4761211)

[SI 4: Reclassification of satellite-based PFTs to LPJmL-PFTs 15](#_Toc4761212)

[SI 5: Sampling of grid cells for the optimization 17](#_Toc4761213)

[References 18](#_Toc4761214)

Figures

[**Supplementary Figure 1:** The optimum parameter set from the tropical optimization experiment causes a bias in herbaceous vegetation cover. 5](#_Toc4761166)

[**Supplementary Figure 2:** Overview of model parameters for productivity and phenology. 6](#_Toc4761167)

[**Supplementary Figure 3:** Overview of model parameters for turnover, mortality and bioclimatic limits. 7](#_Toc4761168)

[**Supplementary Figure 4:** Correlations between selected model parameters from the tropical optimization experiment. 8](#_Toc4761169)

[**Supplementary Figure 5:** Dissimilarity between simulated and observed land cover. 9](#_Toc4761170)

[**Supplementary Figure 6:** Latitudinal gradients of the coverage of different tree types. 10](#_Toc4761171)

[**Supplementary Figure 7:** Dominant plant functional type per grid cell. 10](#_Toc4761172)

[**Supplementary Figure 8:** Evaluation of temporal dynamics in monthly FAPAR, SIF and GPP from LPJmL best model run. 11](#_Toc4761173)

[**Supplementary Figure 10:** Spatial patterns of forest k (yr-1) as the ratio of NPP to biomass as simulated by LPJmL and based oan satellite-derived estimates. 12](#_Toc4761174)

[**Supplementary Figure 11:** Simulated and data-based forest carbon turnover rate (k), NPP and biomass as a function of the number of icing days during a year in boreal forest transects. 13](#_Toc4761175)

[**Supplementary Figure 12:** Simulated and data-based forest carbon turnover rate (k), NPP and biomass as a function of the maximum length of warm-dry periods during a year in temperate forest transects. 14](#_Toc4761176)

[**Supplementary Figure 13:** Maps of PFTs according to the definition of LPJmL derived from the ESA CCI land cover-based PFT map. 16](#_Toc4761177)

[**Supplementary Figure 14:** Map to stratify the sampling of grid cells according to the level of vegetation dynamics. 18](#_Toc4761178)

Tables

**Supplementary Table 1:** Description of LPJmL4 model parameters that were optimized in this study. 3

**Supplementary Table 2:** Best values of each parameter after optimization. 4

**Supplementary Table 3:** Properties of the used satellite-derived datasets 15

**Supplementary Table 4:** Conversion of PFTs from the satellite-based PFT map to LPJmL PFTs according to Köppen-Geiger climate zones. 15

# SI 1: LPJmL model parameters and optimization results

**Supplementary Table 1:** Description of LPJmL4 model parameters that were optimized in this study.

| **Parameter** | **Description** | **Unit** | **Reference** |
| --- | --- | --- | --- |
| **Phenology** |  |  |  |
| LIGHT_BASE | Inflection point of light-limiting function for phenology | W m-2 | Tables S1 and S5 in Forkel et al.^1^ |
| LIGHT_SLOPE | Slope of light-limiting function for phenology | 1/(W m2) | Tables S1 and S5 in Forkel et al.^1^ |
| TMAX_BASE | Inflection point of heat-limiting function for phenology | °C | Tables S1 and S5 in Forkel et al.^1^ |
| TMAX_SLOPE | Slope of heat-limiting function for phenology | 1/°C | Tables S1 and S5 in Forkel et al.^1^ |
| TMIN_BASE | Inflection point of cold-limiting function for phenology | °C | Tables S1 and S5 in Forkel et al.^1^ |
| TMIN_SLOPE | Slope of cold-limiting function for phenology | 1/°C | Tables S1 and S5 in Forkel et al.^1^ |
| WSCAL_BASE | Inflection point of water-limiting function for phenology | [fraction] | Tables S1 and S5 in Forkel et al.^1^ |
| WSCAL_SLOPE | Slope of water-limiting function for phenology | 1/[fraction] | Tables S1 and S5 in Forkel et al.^1^ |
| **Photosynthesis** |  |  |  |
| ALPHAA | Scaling parameter to scale leaf-level photosynthesis to canopy level | - | Table S15 in Schaphoff et al. ^2^ |
| BETA_ROOT | Root distribution slope parameter for water availability | - | Table S8 in Schaphoff et al. ^2^ |
| LIGHTEXTCOEFF | Light extinction coefficient in Lambert-Beer relationship | - | Table S5 in Schaphoff et al.^2^ |
| SIF_A, SIF_B | Parameter in SIF-GPP relationship:  SIF = SIF_A * GPP + SIF_B |  | MacBean et al.^3^ |
| TEMP_PHOTOS_HIGH | Upper limit for temperature optimum for photosynthesis | °C | Table S13 in Schaphoff et al.^2^ |
| TEMP_PHOTOS_LOW | Lower limit for temperature optimum for photosynthesis | °C | Table S13 in Schaphoff et al.^2^ |
| **Turnover** |  |  |  |
| LONGEVITY | Leaf longevity | yr | Table S8 in Schaphoff et al.^2^ |
| TURNOVER_SAPWOOD | Turnover time of sapwood to heartwood | yr | Table S8 in Schaphoff et al.^2^ |
| TURNOVER_LEAF | Turnover time of leafs | yr | Table S8 in Schaphoff et al.^2^ |
| **Establishment and mortality** |  |  |  |
| CROWN_MORT_RCK | Crown damage parameter during fire spread | - | Tables S9 and S15 in Schaphoff et al.^2^ |
| K_EST | Establishment rate | Sapling m-2 | Table S6 in Schaphoff et al.^2^ |
| MORT_MAX | Asymptotic maximum mortality rate | 1/yr | Table S6 in Schaphoff et al.^2^ |
| SCORCHHEIGHT_F | Parameter for fire scorch height | - | Table S9 in Schaphoff et al.^2^ |
| TWMAX_DAILY | Parameter for heat damage function | °C | Table S6 in Schaphoff et al.^2^ |
| **Bioclimatic limits** |  |  |  |
| GDD5MIN | Minimum number of growing degree days (base 5°C) for establishment | °C | Table S4 in Schaphoff et al.^2^ |
| MIN_TEMPRANGE | Minimum temperature amplitude for survival | K | Table S4 in Schaphoff et al.^2^ |
| TEMP_HIGH | Maximum temperature of the coldest month | °C | Table S4 in Schaphoff et al.^2^ |
| TEMP_LOW | Minimum temperature of the coldest month | °C | Table S4 in Schaphoff et al.^2^ |

**Supplementary Table 2:** Prior and best values and lower and upper boundaries of each parameter.

Note that the “best” parameter values for the tropical PFTs are not the values of the optimum parameter set but the values of the parameter set that was manually selected to avoid the bias in herbaceous cover.

| **PFT** | **Parameter name** | **Prior** | **Lower** | **Upper** | **Best** |
| --- | --- | --- | --- | --- | --- |
| BoBS | ALPHAA_BoBS | 0.400 | 0.390 | 0.650 | 0.444 |
| BoNE | ALPHAA_BoNE | 0.400 | 0.390 | 0.650 | 0.440 |
| BoNS | ALPHAA_BoNS | 0.400 | 0.390 | 0.650 | 0.501 |
| PoH | ALPHAA_PoH | 0.400 | 0.390 | 0.650 | 0.519 |
| TeBE | ALPHAA_TeBE | 0.400 | 0.390 | 0.650 | 0.425 |
| TeBS | ALPHAA_TeBS | 0.400 | 0.390 | 0.650 | 0.541 |
| TeH | ALPHAA_TeH | 0.400 | 0.390 | 0.650 | 0.473 |
| TeNE | ALPHAA_TeNE | 0.400 | 0.390 | 0.650 | 0.529 |
| TrBE | ALPHAA_TrBE | 0.400 | 0.390 | 0.650 | 0.437 |
| TrBR | ALPHAA_TrBR | 0.400 | 0.390 | 0.650 | 0.415 |
| TrH | ALPHAA_TrH | 0.400 | 0.390 | 0.650 | 0.421 |
| BoBS | BETA_ROOT_BoBS | 0.943 | 0.940 | 0.990 | 0.940 |
| BoNE | BETA_ROOT_BoNE | 0.943 | 0.940 | 0.990 | 0.940 |
| BoNS | BETA_ROOT_BoNS | 0.943 | 0.940 | 0.990 | 0.959 |
| PoH | BETA_ROOT_PoH | 0.943 | 0.940 | 0.990 | 0.986 |
| TeBE | BETA_ROOT_TeBE | 0.964 | 0.950 | 0.990 | 0.964 |
| TeBS | BETA_ROOT_TeBS | 0.966 | 0.950 | 0.990 | 0.970 |
| TeH | BETA_ROOT_TeH | 0.943 | 0.940 | 0.990 | 0.968 |
| TeNE | BETA_ROOT_TeNE | 0.976 | 0.950 | 0.990 | 0.986 |
| TrBE | BETA_ROOT_TrBE | 0.962 | 0.950 | 0.990 | 0.979 |
| TrBR | BETA_ROOT_TrBR | 0.961 | 0.950 | 0.990 | 0.975 |
| TrH | BETA_ROOT_TrH | 0.972 | 0.950 | 0.990 | 0.959 |
| BoBS | CROWN_MORT_RCK_BoBS | 1.000 | 0.500 | 1.010 | 0.757 |
| BoNE | CROWN_MORT_RCK_BoNE | 1.000 | 0.500 | 1.010 | 0.641 |
| BoNS | CROWN_MORT_RCK_BoNS | 1.000 | 0.500 | 1.010 | 0.761 |
| TeBE | CROWN_MORT_RCK_TeBE | 0.950 | 0.500 | 1.010 | 0.599 |
| TeBS | CROWN_MORT_RCK_TeBS | 1.000 | 0.500 | 1.010 | 0.560 |
| TeNE | CROWN_MORT_RCK_TeNE | 1.000 | 0.500 | 1.010 | 1.001 |
| TrBE | CROWN_MORT_RCK_TrBE | 1.000 | 0.500 | 1.010 | 0.778 |
| TrBR | CROWN_MORT_RCK_TrBR | 0.050 | 0.000 | 0.500 | 0.088 |
| BoBS | GDD5MIN_BoBS | 350.000 | 200.000 | 500.000 | 419.540 |
| BoNE | GDD5MIN_BoNE | 600.000 | 450.000 | 750.000 | 458.540 |
| BoNS | GDD5MIN_BoNS | 350.000 | 200.000 | 500.000 | 225.980 |
| TeBE | GDD5MIN_TeBE | 1200.000 | 1000.000 | 1400.000 | 1095.800 |
| TeBS | GDD5MIN_TeBS | 1200.000 | 1000.000 | 1400.000 | 1058.400 |
| TeNE | GDD5MIN_TeNE | 900.000 | 750.000 | 1050.000 | 805.510 |
| BoBS | K_EST_BoBS | 0.120 | 0.100 | 0.150 | 0.138 |
| BoNE | K_EST_BoNE | 0.120 | 0.100 | 0.150 | 0.149 |
| BoNS | K_EST_BoNS | 0.120 | 0.100 | 0.150 | 0.110 |
| TeBE | K_EST_TeBE | 0.120 | 0.100 | 0.150 | 0.131 |
| TeBS | K_EST_TeBS | 0.120 | 0.100 | 0.150 | 0.148 |
| TeNE | K_EST_TeNE | 0.120 | 0.100 | 0.150 | 0.139 |
| TrBE | K_EST_TrBE | 0.120 | 0.100 | 0.150 | 0.142 |
| TrBR | K_EST_TrBR | 0.120 | 0.100 | 0.150 | 0.123 |
| BoBS | LIGHT_BASE_BoBS | 59.780 | 29.780 | 89.780 | 59.982 |
| BoNE | LIGHT_BASE_BoNE | 3.040 | -26.960 | 33.040 | -25.270 |
| BoNS | LIGHT_BASE_BoNS | 130.100 | 100.100 | 160.100 | 146.830 |
| PoH | LIGHT_BASE_PoH | 50.000 | 20.000 | 80.000 | 67.003 |
| TeBE | LIGHT_BASE_TeBE | 39.320 | 9.320 | 69.320 | 44.007 |
| TeBS | LIGHT_BASE_TeBS | 59.780 | 29.780 | 89.780 | 36.907 |
| TeH | LIGHT_BASE_TeH | 75.940 | 45.940 | 105.940 | 70.410 |
| TeNE | LIGHT_BASE_TeNE | 4.872 | -25.128 | 34.872 | -3.976 |
| TrBE | LIGHT_BASE_TrBE | 55.530 | 25.530 | 85.530 | 48.701 |
| TrBR | LIGHT_BASE_TrBR | 13.010 | -16.990 | 43.010 | -8.310 |
| TrH | LIGHT_BASE_TrH | 69.900 | 39.900 | 99.900 | 68.770 |
| BoBS | LIGHT_SLOPE_BoBS | 58.000 | 28.000 | 88.000 | 52.377 |
| BoNE | LIGHT_SLOPE_BoNE | 14.000 | 5.000 | 44.000 | 15.156 |
| BoNS | LIGHT_SLOPE_BoNS | 95.000 | 65.000 | 125.000 | 103.450 |
| PoH | LIGHT_SLOPE_PoH | 23.000 | 5.000 | 53.000 | 7.552 |
| TeBE | LIGHT_SLOPE_TeBE | 18.830 | 5.000 | 48.830 | 11.226 |
| TeBS | LIGHT_SLOPE_TeBS | 58.000 | 28.000 | 88.000 | 71.622 |
| TeH | LIGHT_SLOPE_TeH | 23.000 | 5.000 | 53.000 | 15.690 |
| TeNE | LIGHT_SLOPE_TeNE | 20.000 | 5.000 | 50.000 | 14.884 |
| TrBE | LIGHT_SLOPE_TrBE | 77.170 | 47.170 | 107.170 | 101.670 |
| TrBR | LIGHT_SLOPE_TrBR | 23.000 | 5.000 | 53.000 | 20.460 |
| TrH | LIGHT_SLOPE_TrH | 64.230 | 34.230 | 94.230 | 48.520 |
| BoBS | LIGHTEXTCOEFF_BoBS | 0.500 | 0.400 | 0.600 | 0.455 |
| BoNE | LIGHTEXTCOEFF_BoNE | 0.500 | 0.400 | 0.600 | 0.519 |
| BoNS | LIGHTEXTCOEFF_BoNS | 0.600 | 0.500 | 0.700 | 0.685 |
| PoH | LIGHTEXTCOEFF_PoH | 0.500 | 0.400 | 0.600 | 0.461 |
| TeBE | LIGHTEXTCOEFF_TeBE | 0.500 | 0.400 | 0.600 | 0.560 |
| TeBS | LIGHTEXTCOEFF_TeBS | 0.600 | 0.500 | 0.700 | 0.596 |
| TeH | LIGHTEXTCOEFF_TeH | 0.500 | 0.400 | 0.600 | 0.481 |
| TeNE | LIGHTEXTCOEFF_TeNE | 0.400 | 0.300 | 0.500 | 0.425 |
| TrBE | LIGHTEXTCOEFF_TrBE | 0.500 | 0.400 | 0.600 | 0.530 |
| TrBR | LIGHTEXTCOEFF_TrBR | 0.500 | 0.400 | 0.600 | 0.526 |
| TrH | LIGHTEXTCOEFF_TrH | 0.400 | 0.300 | 0.500 | 0.487 |
| BoBS | LONGEVITY_BoBS | 0.500 | 0.450 | 0.550 | 0.508 |
| BoNE | LONGEVITY_BoNE | 4.000 | 3.000 | 5.000 | 3.242 |
| BoNS | LONGEVITY_BoNS | 0.650 | 0.600 | 0.700 | 0.620 |
| PoH | LONGEVITY_PoH | 0.350 | 0.300 | 0.400 | 0.380 |
| TeBE | LONGEVITY_TeBE | 1.600 | 1.000 | 2.000 | 1.112 |
| TeBS | LONGEVITY_TeBS | 0.450 | 0.400 | 0.500 | 0.465 |
| TeH | LONGEVITY_TeH | 0.350 | 0.300 | 0.400 | 0.314 |
| TeNE | LONGEVITY_TeNE | 4.000 | 3.000 | 5.000 | 3.378 |
| TrBE | LONGEVITY_TrBE | 1.600 | 1.000 | 2.000 | 1.224 |
| TrBR | LONGEVITY_TrBR | 0.500 | 0.450 | 0.550 | 0.528 |
| TrH | LONGEVITY_TrH | 0.400 | 0.350 | 0.450 | 0.387 |
| BoNS | MIN_TEMPRANGE_BoNS | 30.000 | 20.000 | 40.000 | 25.909 |
| PoH | MIN_TEMPRANGE_PoH | 18.000 | 8.000 | 28.000 | 12.231 |
| BoBS | MORT_MAX_BoBS | 0.030 | 0.010 | 0.031 | 0.023 |
| BoNE | MORT_MAX_BoNE | 0.030 | 0.010 | 0.031 | 0.027 |
| BoNS | MORT_MAX_BoNS | 0.030 | 0.010 | 0.031 | 0.018 |
| TeBE | MORT_MAX_TeBE | 0.030 | 0.010 | 0.031 | 0.022 |
| TeBS | MORT_MAX_TeBS | 0.030 | 0.010 | 0.031 | 0.024 |
| TeNE | MORT_MAX_TeNE | 0.030 | 0.010 | 0.031 | 0.018 |
| TrBE | MORT_MAX_TrBE | 0.030 | 0.010 | 0.031 | 0.028 |
| TrBR | MORT_MAX_TrBR | 0.030 | 0.010 | 0.031 | 0.027 |
| BoBS | SCORCHHEIGHT_F_BoBS | 0.094 | 0.047 | 0.141 | 0.122 |
| BoNE | SCORCHHEIGHT_F_BoNE | 0.110 | 0.055 | 0.165 | 0.125 |
| BoNS | SCORCHHEIGHT_F_BoNS | 0.094 | 0.047 | 0.141 | 0.095 |
| TeBE | SCORCHHEIGHT_F_TeBE | 0.371 | 0.186 | 0.557 | 0.375 |
| TeBS | SCORCHHEIGHT_F_TeBS | 0.094 | 0.047 | 0.141 | 0.078 |
| TeNE | SCORCHHEIGHT_F_TeNE | 0.100 | 0.050 | 0.150 | 0.081 |
| TrBE | SCORCHHEIGHT_F_TrBE | 0.149 | 0.074 | 0.223 | 0.096 |
| TrBR | SCORCHHEIGHT_F_TrBR | 0.061 | 0.031 | 0.092 | 0.066 |
| BoBS | SIF_A_BoBS | 0.311 | 0.211 | 0.411 | 0.269 |
| BoNE | SIF_A_BoNE | 0.190 | 0.090 | 0.290 | 0.157 |
| BoNS | SIF_A_BoNS | 0.233 | 0.133 | 0.333 | 0.229 |
| PoH | SIF_A_PoH | 0.469 | 0.369 | 0.569 | 0.528 |
| TeBE | SIF_A_TeBE | 0.216 | 0.116 | 0.569 | 0.122 |
| TeBS | SIF_A_TeBS | 0.278 | 0.178 | 0.378 | 0.289 |
| TeH | SIF_A_TeH | 0.421 | 0.321 | 0.521 | 0.325 |
| TeNE | SIF_A_TeNE | 0.220 | 0.120 | 0.320 | 0.166 |
| TrBE | SIF_A_TrBE | 0.100 | 0.010 | 0.200 | 0.092 |
| TrBR | SIF_A_TrBR | 0.258 | 0.158 | 0.358 | 0.266 |
| TrH | SIF_A_TrH | 0.375 | 0.275 | 0.475 | 0.290 |
| BoBS | SIF_B_BoBS | 0.092 | -0.060 | 0.138 | -0.060 |
| BoNE | SIF_B_BoNE | 0.128 | -0.060 | 0.192 | 0.016 |
| BoNS | SIF_B_BoNS | -0.019 | -0.060 | 0.100 | -0.035 |
| PoH | SIF_B_PoH | -0.043 | -0.060 | 0.100 | -0.033 |
| TeBE | SIF_B_TeBE | 0.445 | -0.060 | 0.668 | -0.022 |
| TeBS | SIF_B_TeBS | 0.404 | -0.060 | 0.606 | 0.044 |
| TeH | SIF_B_TeH | 0.106 | -0.060 | 0.159 | -0.054 |
| TeNE | SIF_B_TeNE | 0.261 | -0.060 | 0.392 | -0.032 |
| TrBE | SIF_B_TrBE | 1.349 | -0.060 | 2.024 | 1.161 |
| TrBR | SIF_B_TrBR | 0.448 | -0.060 | 0.672 | 0.221 |
| TrH | SIF_B_TrH | 0.197 | -0.060 | 0.296 | -0.027 |
| BoBS | TEMP_HIGH_BoBS | -2.000 | -12.000 | 8.000 | 5.774 |
| BoNE | TEMP_HIGH_BoNE | -2.000 | -12.000 | 8.000 | -3.048 |
| BoNS | TEMP_HIGH_BoNS | -5.400 | -15.400 | 4.600 | -15.391 |
| PoH | TEMP_HIGH_PoH | -2.600 | -12.600 | 7.400 | 2.516 |
| TeBE | TEMP_HIGH_TeBE | 18.800 | 8.800 | 28.800 | 16.623 |
| TeBS | TEMP_HIGH_TeBS | 15.500 | 5.500 | 25.500 | 11.454 |
| TeH | TEMP_HIGH_TeH | 15.500 | 5.500 | 25.500 | 5.777 |
| TeNE | TEMP_HIGH_TeNE | 22.000 | 12.000 | 32.000 | 16.024 |
| BoNE | TEMP_LOW_BoNE | -32.500 | -42.500 | -22.500 | -31.978 |
| BoNS | TEMP_LOW_BoNS | -46.000 | -56.000 | -36.000 | -55.363 |
| TeBE | TEMP_LOW_TeBE | 3.000 | -7.000 | 13.000 | 1.189 |
| TeBS | TEMP_LOW_TeBS | -17.700 | -27.700 | -7.700 | -22.858 |
| TeH | TEMP_LOW_TeH | -39.000 | -49.000 | -29.000 | -48.170 |
| TeNE | TEMP_LOW_TeNE | -2.000 | -12.000 | 8.000 | -6.325 |
| TrBE | TEMP_LOW_TrBE | 15.500 | 5.500 | 25.500 | 18.400 |
| TrBR | TEMP_LOW_TrBR | 15.500 | 5.500 | 25.500 | 13.380 |
| TrH | TEMP_LOW_TrH | 7.000 | -3.000 | 17.000 | 15.210 |
| BoBS | TEMP_PHOTOS_HIGH_BoBS | 25.000 | 20.000 | 30.000 | 27.878 |
| BoNE | TEMP_PHOTOS_HIGH_BoNE | 25.000 | 20.000 | 30.000 | 21.925 |
| BoNS | TEMP_PHOTOS_HIGH_BoNS | 25.000 | 20.000 | 30.000 | 24.012 |
| PoH | TEMP_PHOTOS_HIGH_PoH | 30.000 | 25.000 | 35.000 | 25.195 |
| TeBE | TEMP_PHOTOS_HIGH_TeBE | 30.000 | 25.000 | 35.000 | 33.153 |
| TeBS | TEMP_PHOTOS_HIGH_TeBS | 25.000 | 20.000 | 30.000 | 25.257 |
| TeH | TEMP_PHOTOS_HIGH_TeH | 30.000 | 25.000 | 35.000 | 28.231 |
| TeNE | TEMP_PHOTOS_HIGH_TeNE | 30.000 | 25.000 | 35.000 | 31.794 |
| TrBE | TEMP_PHOTOS_HIGH_TrBE | 30.000 | 25.000 | 35.000 | 30.185 |
| TrBR | TEMP_PHOTOS_HIGH_TrBR | 30.000 | 25.000 | 35.000 | 32.010 |
| TrH | TEMP_PHOTOS_HIGH_TrH | 45.000 | 40.000 | 50.000 | 44.340 |
| BoBS | TEMP_PHOTOS_LOW_BoBS | 15.000 | 10.000 | 20.000 | 18.520 |
| BoNE | TEMP_PHOTOS_LOW_BoNE | 15.000 | 10.000 | 20.000 | 17.695 |
| BoNS | TEMP_PHOTOS_LOW_BoNS | 15.000 | 10.000 | 20.000 | 18.267 |
| PoH | TEMP_PHOTOS_LOW_PoH | 10.000 | 5.000 | 15.000 | 6.362 |
| TeBE | TEMP_PHOTOS_LOW_TeBE | 20.000 | 15.000 | 25.000 | 16.170 |
| TeBS | TEMP_PHOTOS_LOW_TeBS | 20.000 | 15.000 | 25.000 | 22.210 |
| TeH | TEMP_PHOTOS_LOW_TeH | 10.000 | 5.000 | 15.000 | 10.675 |
| TeNE | TEMP_PHOTOS_LOW_TeNE | 20.000 | 15.000 | 25.000 | 21.806 |
| TrBE | TEMP_PHOTOS_LOW_TrBE | 25.000 | 20.000 | 30.000 | 27.899 |
| TrBR | TEMP_PHOTOS_LOW_TrBR | 25.000 | 20.000 | 30.000 | 26.560 |
| TrH | TEMP_PHOTOS_LOW_TrH | 20.000 | 15.000 | 25.000 | 18.180 |
| BoBS | TMAX_BASE_BoBS | 41.510 | 21.510 | 61.510 | 36.914 |
| BoNE | TMAX_BASE_BoNE | 27.320 | 7.320 | 47.320 | 35.230 |
| BoNS | TMAX_BASE_BoNS | 44.600 | 24.600 | 64.600 | 31.788 |
| PoH | TMAX_BASE_PoH | 26.120 | 6.120 | 46.120 | 8.523 |
| TeBE | TMAX_BASE_TeBE | 41.120 | 21.120 | 61.120 | 28.467 |
| TeBS | TMAX_BASE_TeBS | 41.510 | 21.510 | 61.510 | 24.231 |
| TeH | TMAX_BASE_TeH | 32.040 | 12.040 | 52.040 | 35.431 |
| TeNE | TMAX_BASE_TeNE | 35.260 | 15.260 | 55.260 | 42.407 |
| TrBE | TMAX_BASE_TrBE | 38.640 | 18.640 | 58.640 | 53.740 |
| TrBR | TMAX_BASE_TrBR | 38.640 | 18.640 | 58.640 | 36.650 |
| TrH | TMAX_BASE_TrH | 29.160 | 9.160 | 49.160 | 25.230 |
| BoBS | TMAX_SLOPE_BoBS | 1.740 | 0.740 | 2.740 | 2.597 |
| BoNE | TMAX_SLOPE_BoNE | 0.240 | 0.100 | 1.240 | 0.379 |
| BoNS | TMAX_SLOPE_BoNS | 0.240 | 0.100 | 1.240 | 0.567 |
| PoH | TMAX_SLOPE_PoH | 0.240 | 0.100 | 1.240 | 0.357 |
| TeBE | TMAX_SLOPE_TeBE | 0.980 | 0.100 | 1.980 | 1.110 |
| TeBS | TMAX_SLOPE_TeBS | 1.740 | 0.740 | 2.740 | 1.703 |
| TeH | TMAX_SLOPE_TeH | 0.240 | 0.100 | 1.240 | 0.507 |
| TeNE | TMAX_SLOPE_TeNE | 1.830 | 0.830 | 2.830 | 2.740 |
| TrBE | TMAX_SLOPE_TrBE | 1.860 | 0.860 | 2.860 | 2.556 |
| TrBR | TMAX_SLOPE_TrBR | 1.625 | 0.625 | 2.625 | 1.951 |
| TrH | TMAX_SLOPE_TrH | 1.470 | 0.470 | 2.470 | 2.330 |
| BoBS | TMIN_BASE_BoBS | 2.045 | -2.955 | 7.045 | 0.112 |
| BoNE | TMIN_BASE_BoNE | -7.516 | -12.516 | -2.516 | -10.043 |
| BoNS | TMIN_BASE_BoNS | -4.165 | -9.165 | 0.835 | -8.692 |
| PoH | TMIN_BASE_PoH | 2.790 | -2.210 | 7.790 | -0.574 |
| TeBE | TMIN_BASE_TeBE | -0.630 | -5.630 | 4.370 | 0.996 |
| TeBS | TMIN_BASE_TeBS | 13.690 | 8.690 | 18.690 | 10.116 |
| TeH | TMIN_BASE_TeH | 4.979 | -0.021 | 9.979 | 8.321 |
| TeNE | TMIN_BASE_TeNE | -7.813 | -12.813 | -2.813 | -8.880 |
| TrBE | TMIN_BASE_TrBE | 8.300 | 3.300 | 13.300 | 10.085 |
| TrBR | TMIN_BASE_TrBR | 7.660 | 2.660 | 12.660 | 11.960 |
| TrH | TMIN_BASE_TrH | 6.418 | 1.418 | 11.418 | 8.052 |
| BoBS | TMIN_SLOPE_BoBS | 0.215 | 0.015 | 0.415 | 0.101 |
| BoNE | TMIN_SLOPE_BoNE | 0.101 | -0.099 | 0.301 | 0.047 |
| BoNS | TMIN_SLOPE_BoNS | 0.150 | -0.050 | 0.350 | 0.084 |
| PoH | TMIN_SLOPE_PoH | 0.130 | -0.070 | 0.330 | 0.103 |
| TeBE | TMIN_SLOPE_TeBE | 0.550 | 0.350 | 0.750 | 0.471 |
| TeBS | TMIN_SLOPE_TeBS | 0.259 | 0.059 | 0.459 | 0.255 |
| TeH | TMIN_SLOPE_TeH | 0.311 | 0.111 | 0.511 | 0.191 |
| TeNE | TMIN_SLOPE_TeNE | 0.217 | 0.017 | 0.417 | 0.070 |
| TrBE | TMIN_SLOPE_TrBE | 1.010 | 0.810 | 1.210 | 1.007 |
| TrBR | TMIN_SLOPE_TrBR | 0.240 | 0.040 | 0.440 | 0.156 |
| TrH | TMIN_SLOPE_TrH | 0.910 | 0.710 | 1.110 | 0.886 |
| BoBS | TURNOVER_LEAF_BoBS | 1.000 | 0.500 | 1.500 | 0.890 |
| BoNE | TURNOVER_LEAF_BoNE | 4.000 | 2.000 | 6.000 | 5.178 |
| BoNS | TURNOVER_LEAF_BoNS | 1.000 | 0.500 | 1.500 | 1.008 |
| PoH | TURNOVER_LEAF_PoH | 1.000 | 0.500 | 1.500 | 0.785 |
| TeBE | TURNOVER_LEAF_TeBE | 1.000 | 0.500 | 1.500 | 1.432 |
| TeBS | TURNOVER_LEAF_TeBS | 1.000 | 0.500 | 1.500 | 0.546 |
| TeH | TURNOVER_LEAF_TeH | 1.000 | 0.500 | 1.500 | 0.997 |
| TeNE | TURNOVER_LEAF_TeNE | 4.000 | 2.000 | 6.000 | 3.556 |
| TrBE | TURNOVER_LEAF_TrBE | 2.000 | 1.000 | 3.000 | 1.885 |
| TrBR | TURNOVER_LEAF_TrBR | 1.000 | 0.500 | 1.500 | 1.222 |
| TrH | TURNOVER_LEAF_TrH | 1.000 | 0.500 | 1.500 | 1.054 |
| BoBS | TURNOVER_SAPWOOD_BoBS | 20.000 | 15.000 | 30.000 | 23.105 |
| BoNE | TURNOVER_SAPWOOD_BoNE | 20.000 | 15.000 | 30.000 | 26.638 |
| BoNS | TURNOVER_SAPWOOD_BoNS | 20.000 | 15.000 | 30.000 | 28.503 |
| TeBE | TURNOVER_SAPWOOD_TeBE | 20.000 | 15.000 | 30.000 | 23.707 |
| TeBS | TURNOVER_SAPWOOD_TeBS | 20.000 | 15.000 | 30.000 | 27.533 |
| TeNE | TURNOVER_SAPWOOD_TeNE | 20.000 | 15.000 | 30.000 | 27.179 |
| TrBE | TURNOVER_SAPWOOD_TrBE | 20.000 | 15.000 | 30.000 | 27.488 |
| TrBR | TURNOVER_SAPWOOD_TrBR | 20.000 | 15.000 | 30.000 | 26.310 |
| BoBS | TWMAX_DAILY_BoBS | 25.000 | 20.000 | 30.000 | 26.533 |
| BoNE | TWMAX_DAILY_BoNE | 25.000 | 20.000 | 30.000 | 22.688 |
| BoNS | TWMAX_DAILY_BoNS | 25.000 | 20.000 | 30.000 | 23.116 |
| BoBS | WSCAL_BASE_BoBS | 20.960 | 0.960 | 40.960 | 25.763 |
| BoNE | WSCAL_BASE_BoNE | 0.008 | -19.992 | 20.008 | -3.210 |
| BoNS | WSCAL_BASE_BoNS | 2.344 | -17.656 | 22.344 | -1.562 |
| PoH | WSCAL_BASE_PoH | 1.000 | -19.000 | 21.000 | -16.270 |
| TeBE | WSCAL_BASE_TeBE | 8.821 | -11.179 | 28.821 | -0.663 |
| TeBS | WSCAL_BASE_TeBS | 20.960 | 0.960 | 40.960 | 35.038 |
| TeH | WSCAL_BASE_TeH | 53.070 | 33.070 | 73.070 | 48.403 |
| TeNE | WSCAL_BASE_TeNE | 8.613 | -11.387 | 28.613 | 1.414 |
| TrBE | WSCAL_BASE_TrBE | 4.997 | -15.003 | 24.997 | 8.848 |
| TrBR | WSCAL_BASE_TrBR | 22.210 | 2.210 | 42.210 | 11.990 |
| TrH | WSCAL_BASE_TrH | 41.720 | 21.720 | 61.720 | 45.910 |
| BoBS | WSCAL_SLOPE_BoBS | 5.240 | 2.240 | 8.240 | 7.386 |
| BoNE | WSCAL_SLOPE_BoNE | 5.000 | 2.000 | 8.000 | 6.713 |
| BoNS | WSCAL_SLOPE_BoNS | 5.000 | 2.000 | 8.000 | 4.200 |
| PoH | WSCAL_SLOPE_PoH | 0.880 | 0.010 | 3.880 | 1.726 |
| TeBE | WSCAL_SLOPE_TeBE | 5.000 | 2.000 | 8.000 | 3.859 |
| TeBS | WSCAL_SLOPE_TeBS | 5.240 | 2.240 | 8.240 | 7.486 |
| TeH | WSCAL_SLOPE_TeH | 0.522 | 0.010 | 3.522 | 2.278 |
| TeNE | WSCAL_SLOPE_TeNE | 5.000 | 2.000 | 8.000 | 2.593 |
| TrBE | WSCAL_SLOPE_TrBE | 5.140 | 2.140 | 8.140 | 4.438 |
| TrBR | WSCAL_SLOPE_TrBR | 7.970 | 4.970 | 10.970 | 7.708 |
| TrH | WSCAL_SLOPE_TrH | 0.100 | 0.010 | 3.100 | 1.314 |


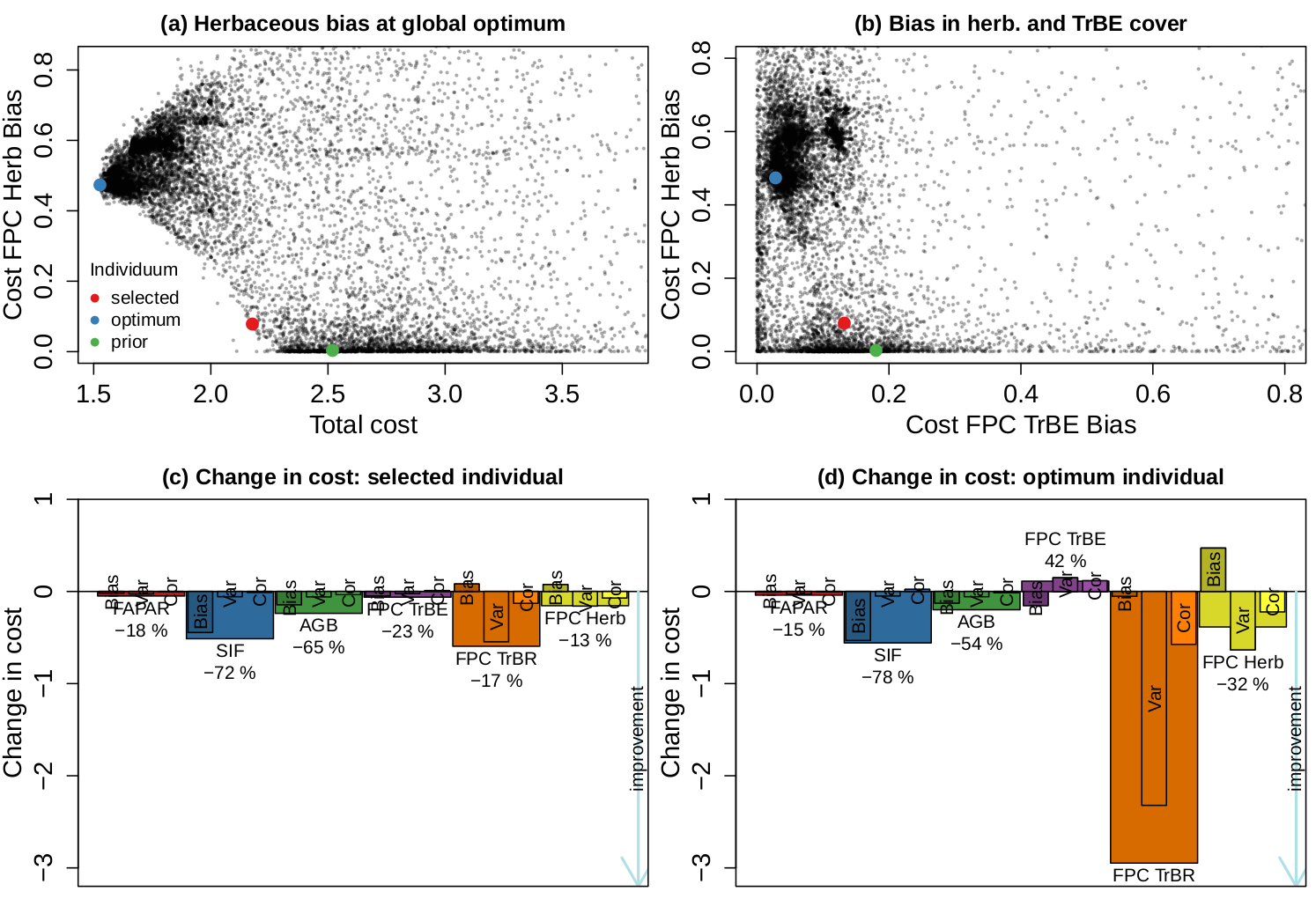


**Supplementary Figure 1:** The optimum parameter set from the tropical optimization experiment causes a bias in herbaceous vegetation cover.

Panels (a) and (b) show the cost component by the bias in herbaceous vegetation cover from all individual parameter sets from the tropical optimization experiment against the total cost (a) and against the cost component by the bias in the cover of tropical broad-leaved evergreen trees. Panels (c) and (d) show the change in cost components between the prior parameter set and the selected alternative best parameter set (red dot in a and b) and the optimum parameter set (blue dot in a and b).


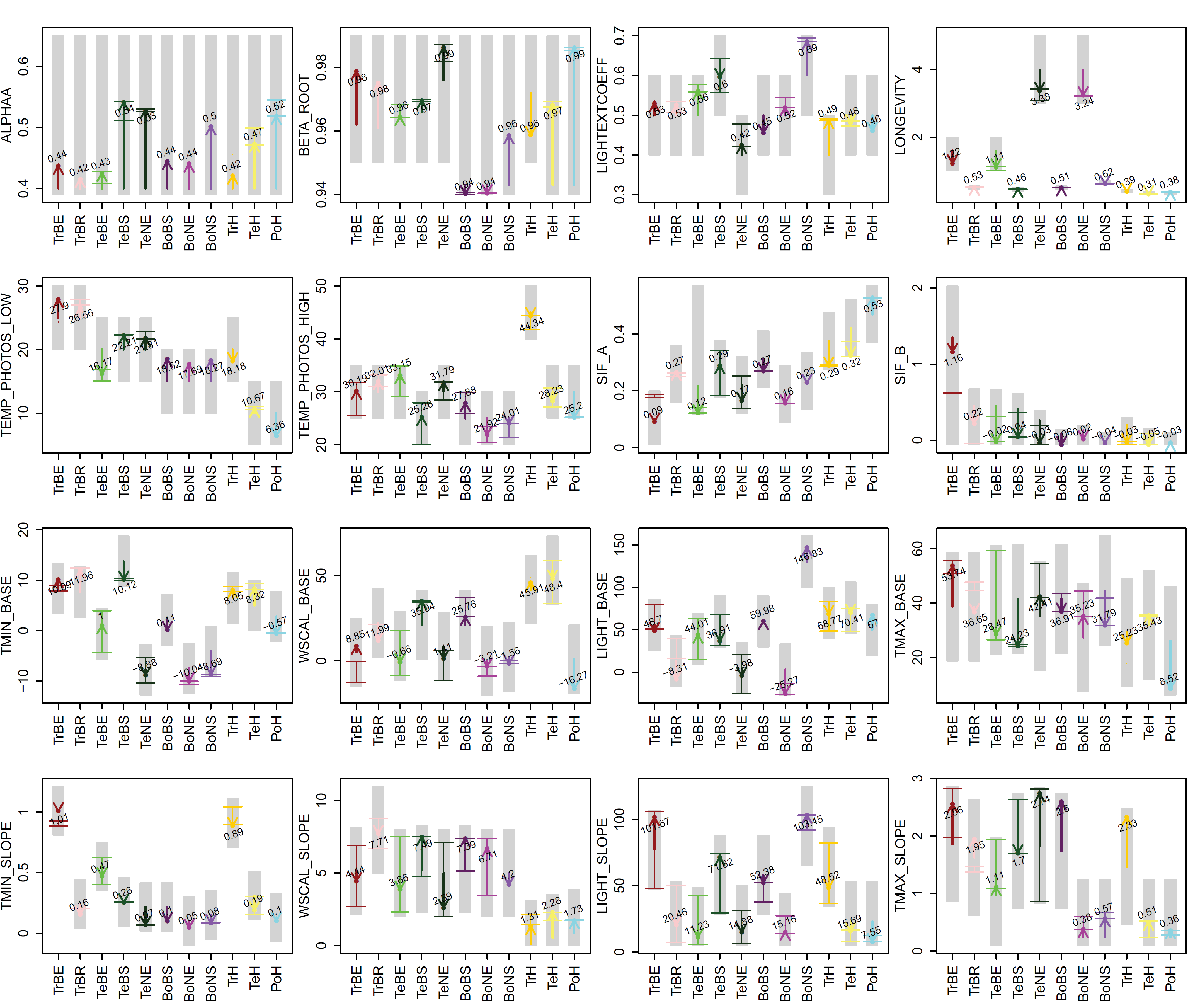


**Supplementary Figure 2:** Overview of model parameters for productivity and phenology.

Grey bars are the uniform prior parameter ranges, arrows show the change from the prior to the “best” parameter values and coloured error bars are the posterior parameter uncertainty (defined as the range of a parameter across all parameter sets with the lowest 5 % of the cost). Note that the “best” parameter values for tropical PFTs are not the values of the optimum parameter set but the values of the parameter set that was manually selected to avoid the bias in herbaceous cover. Hence, the posterior parameter uncertainties for the tropical PFTs are not associated with the “best” parameter.


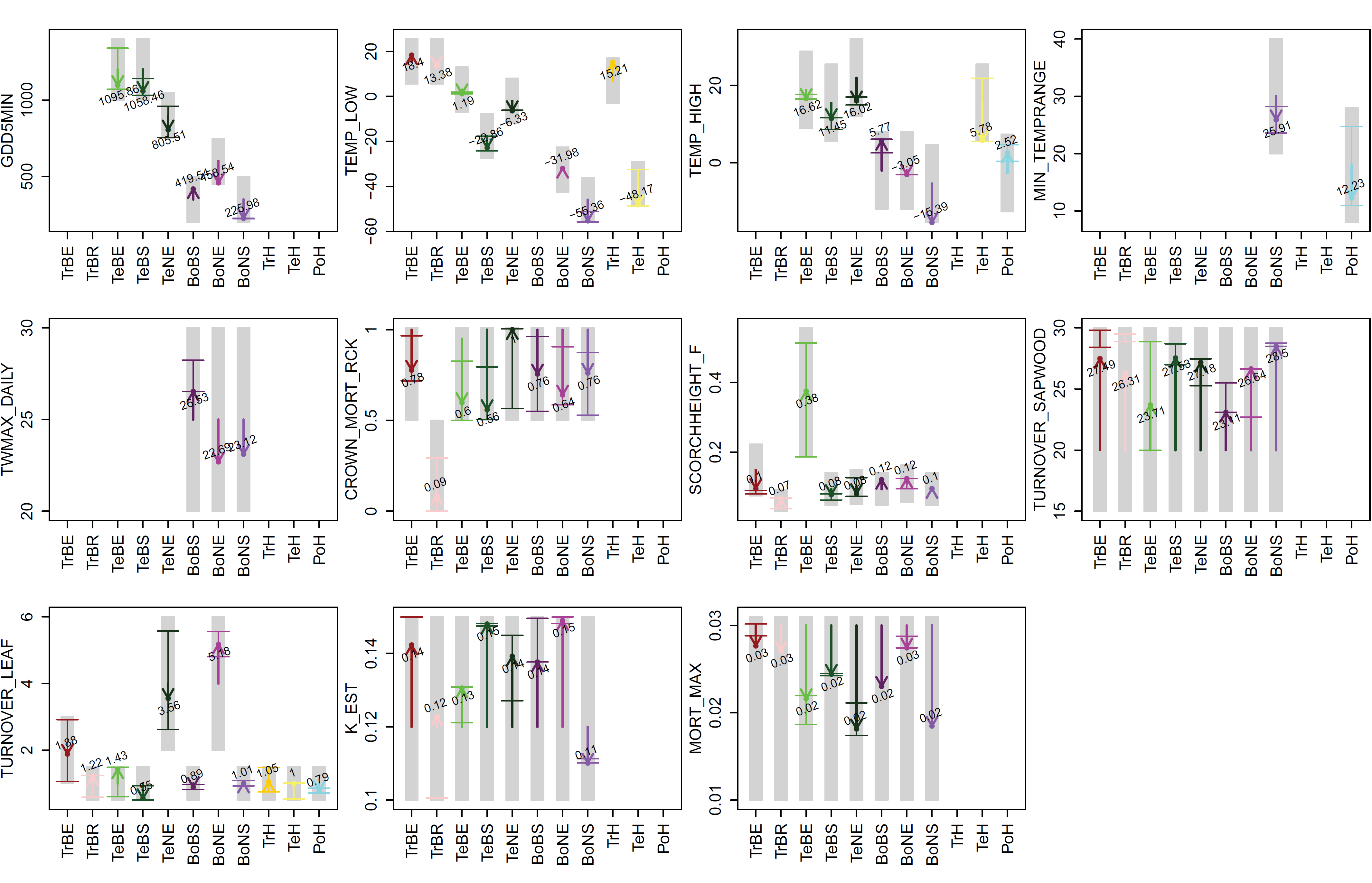


**Supplementary Figure 3:** Overview of model parameters for turnover, mortality and bioclimatic limits.

Grey bars are the uniform prior parameter ranges, arrows show the change from the prior to the “best” parameter values and coloured error bars are the posterior parameter uncertainty (defined as the range of a parameter across all parameter sets with the lowest 5 % of the cost). Note that the “best” parameter values for tropical PFTs are not the values of the optimum parameter set but the values of the parameter set that was manually selected to avoid the bias in herbaceous cover. Hence, the posterior parameter uncertainties for the tropical PFTs are not associated with the “best” parameter.

**Supplementary Figure 4:** Correlations between selected model parameters from the tropical optimization experiment.

Shown are also the total cost (second last column/row) and the cost component from the bias in herbaceous vegetation cover (last column/row). Correlations are based on the Spearman rank-correlation coefficient by using all ~24,000 iterations of this optimization experiment. Only the parameters are shown that had an absolute correlation > 0.2 with the bias component for herbaceous vegetation cover.

# SI 2: Global model evaluation

After the three optimization experiments, we used the best-performing parameter sets from each zone to make a global model run and to evaluate model results for all global grid cells (excluding agricultrual areas, i.e. cropland cover > 20%). We evaluated the global model results against the same datasets (FAPAR, SIF, AGB, land cover) and additionally against GPP. We computed the correlation between simulated and observed FAPAR, SIF and GPP time series to evaluate temporal dynamics. To evaluate absolute changes in GPP, tree cover, and biomass, we also computed mean annual total GPP, mean tree cover (i.e. sum of all tree PFTs), and mean tree AGB and computed the absolute average error (aAE) for each grid cell:

$aAE=|\bar{s}-\bar{o}|$ (1)

We evaluated the simulated land cover fractions by computing the grid cell-level dissimilarity between the simulated and observed fractions of each land cover type. According to Poulter et al. ^4^ the dissimilarity between two sets of PFT fractions can be calculated as the Euclidean distance D between the observed and simulated foliar projective cover of each land cover type:

$D=\sqrt{\sum_{t=1}^{N} \left( FPC_{t,s}-FPC_{t,o} \right)^{2}}$ (2)

Where t {1…N} are the land cover types needle-leaved evergreen trees, needle-leaved deciduous trees, broadleaved evergreen trees, broadleaved deciduous trees, and herbaceous cover. The dissimilarity between simulated and observed land cover is shown in **Supplementary Figure 5**.


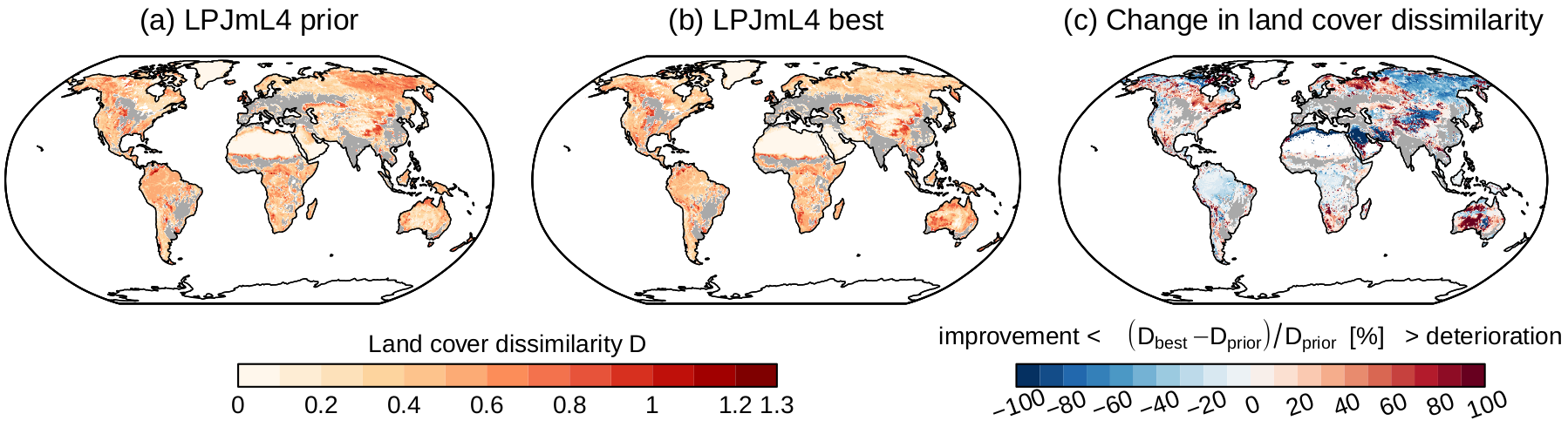


**Supplementary Figure 5:** Dissimilarity between simulated and observed land cover.

Panels (a) and (b) show the dissimilarity from the LPJmL4 prior and best model runs. Panel (c) shows the percentage change in dissimilarity. Blue colours indicate improvements in the simulated land cover fractions in comparison to the observations.


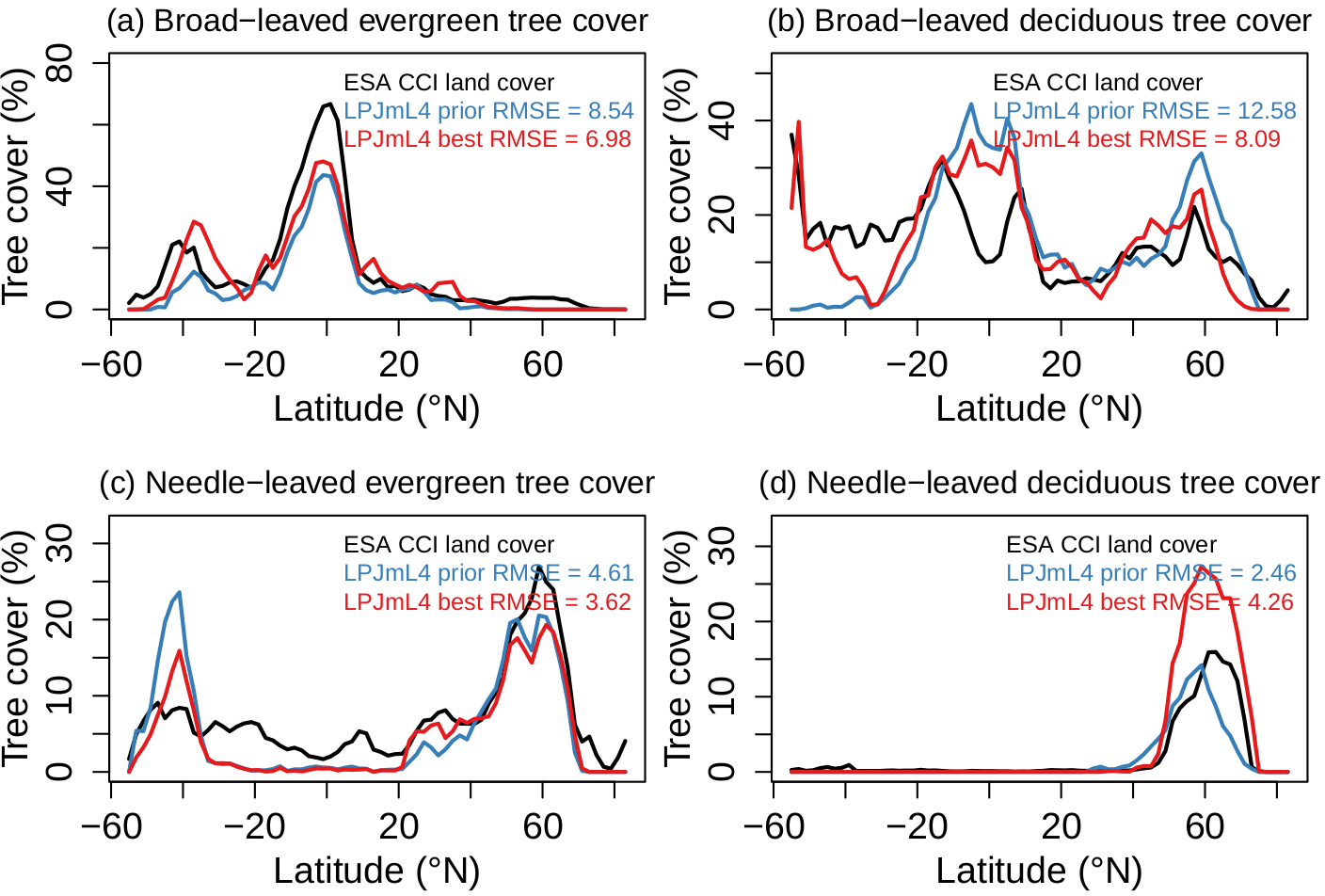


**Supplementary Figure 6:** Latitudinal gradients of the coverage of different tree types.


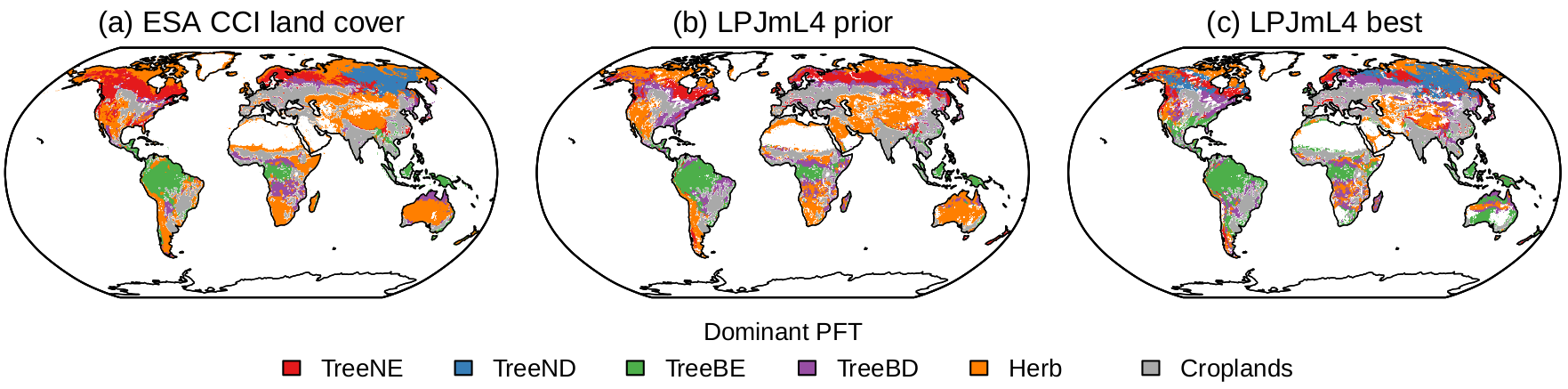


**Supplementary Figure 7:** Dominant plant functional type per grid cell.

The dominant land cover type is here simply the PFT with the maximum coverage.


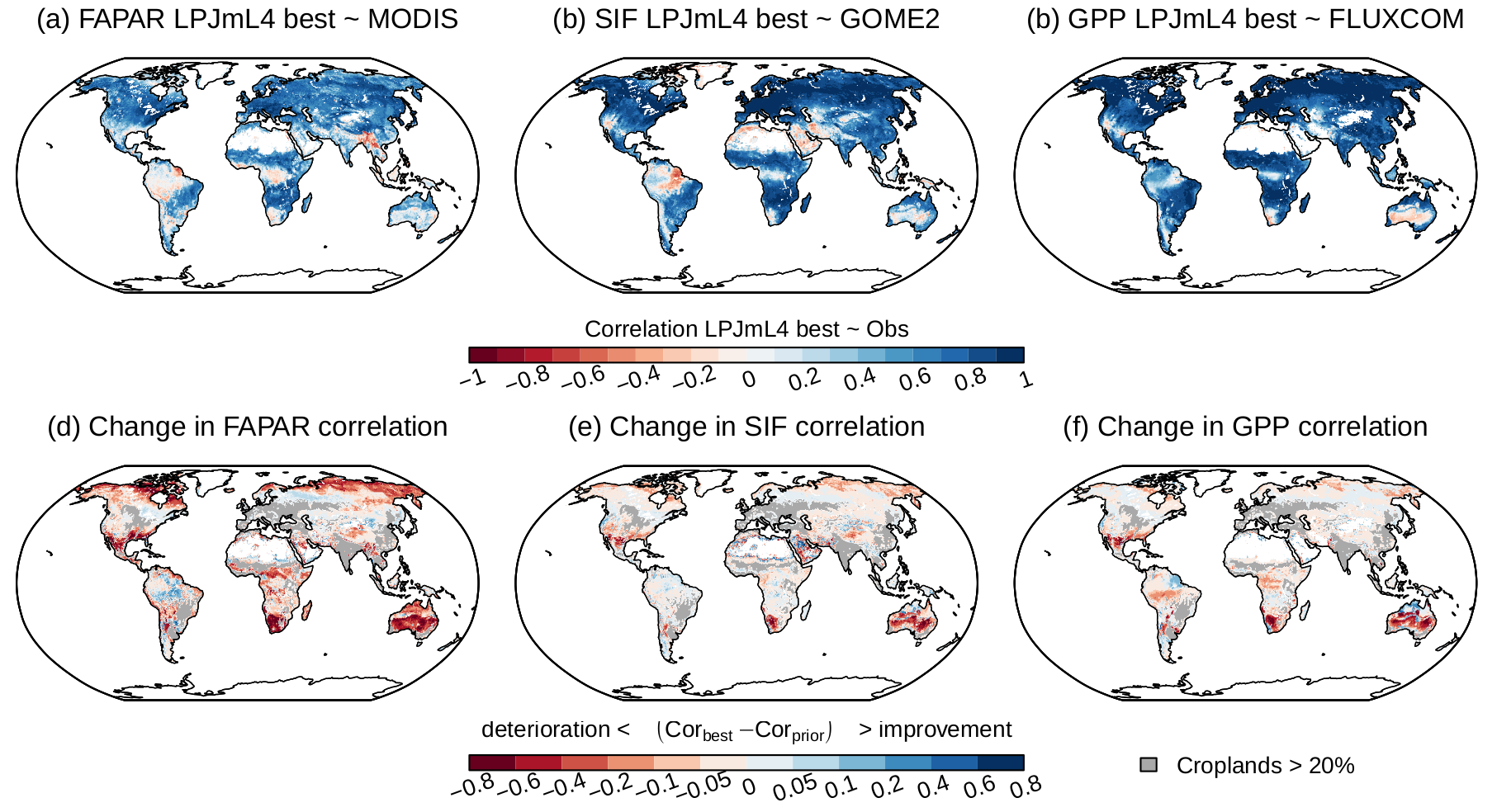


**Supplementary Figure 8:** Evaluation of temporal dynamics in monthly FAPAR, SIF and GPP from LPJmL best model run.

Panels (a-c) show correlations between simulations and observations for the respective overlapping periods between data and models. Panels (d-f) show changes in the correlation between the LPJmL-prior and -best model runs.

# SI 3: Regional relations between climate and forest C turnover rates

In our previous work, we used satellite-based estimates of forest biomass and net primary production (NPP) to assess relationships between climate and forest carbon turnover rates in northern temperate and boreal forest^5,6^. We here repeated these analyses with results from the LPJmL-prior and -best model runs. Forest carbon turnover rate *k* (year^-1^, the inverse of the turnover time τ) is defined as:

$k=\frac{NPP}{VegC}$ (3)

Likewise in our previous work ^5,6^, we used two satellite-derived datasets of NPP (MODIS C5 NPP and BETHY/DLR) and the biomass maps for northern forests^7^ to calculate *k*. As the forest biomass map is based on the GLC2000 land cover maps but we here used the more recent ESA CCI land cover maps to optimize LPJmL, we calculate *k* only for grid cells were both maps show >40% forest cover. We found that LPJmL underestimated the forest carbon turnover rate in large parts of boreal and temperate forests (**Supplementary Figure 9**). LPJmL-best slightly improved over LPJmL-prior in western Norther America and in central Siberia. We then analysed *k*, NPP and forest biomass in relation to the number of icing days per year (i.e. annual number of days with daily maximum temperature < 0°C) and the maximum length of warm-dry periods (i.e. consecutive days with Tmax >= 10°C and without precipitation). Regional relationships are derived by fitting smoothing splines for different transects. Although the optimized model had smaller biases in simulated biomass than the prior model, we found no improvements in the shape of the relationship between *k* and the number of icing days (**Supplementary Figure 10**) or the length of warm-dry periods (**Supplementary Figure 11**).

**Supplementary Figure 9:** Spatial patterns of forest k (yr-1) as the ratio of NPP to biomass as simulated by LPJmL and based oan satellite-derived estimates.

Only areas with at least 40% forest cover are shown. Red boxes show selected transects as in Thurner et al. ^5,6^ (b1 Canada, b2 Western Russia, b3 Central Siberia, b4 eastern Siberia, t1 western North America, t2 south-eastern North America, t3 south-western Europe, t4 north-eastern China/Korea).

**Supplementary Figure 10:** Simulated and data-based forest carbon turnover rate (k), NPP and biomass as a function of the number of icing days during a year in boreal forest transects.

The type of figure and transects (shown in **Supplementary Figure 9**) are according to Thurner et al.^6^.

**Supplementary Figure 11:** Simulated and data-based forest carbon turnover rate (k), NPP and biomass as a function of the maximum length of warm-dry periods during a year in temperate forest transects.

The type of figure and transects (shown in **Supplementary Figure 9**) are according to Thurner et al.^6^.

# SI 4: Reclassification of satellite-based PFTs to LPJmL-PFTs

**Supplementary Table 3:** Properties of the used satellite-derived datasets

| **Variable** | **Dataset, satellite sensor, or**  **algorithm** | **Used temporal coverage** | **Use of dataset (footnotes)** | **Reference** |
| --- | --- | --- | --- | --- |
| FAPAR | MODIS (MOD15A2) | 2000-2015 | O, E | ^8^ |
| SIF | GOME-2 (GlobFluo) | 2007-2014 | O, E | ^9^ |
| AGB  (tropics) | Avitabile et al. (2016) | ~2000-2010  (LPJmL: 2009-2011) | O, E | ^10^ |
| AGB (temperate, boreal) | BIOMASAR (Thurner et al. 2014) | ~2010  (LPJmL: 2009-2011) | O, E | ^7^ |
| PFT cover | PFT maps derived from ESA Land cover_cci V2.0.7 | 1992-2015 | O, E | ^11^ |
| Burned area | ESA Fire_cci V50  (MODIS) | 2001-2016 | P | ^12^ |
| GPP | FLUXCOM (RS+METEO) | 1982-2010 | E | ^13^ |

O: Dataset was used within the cost function for model *O*ptimization. E: Dataset was used for the *E*valuation of global model results. P: Burned area was directly *P*rescribed to the LPJmL4 fire module.

**Supplementary Table 4:** Conversion of PFTs from the satellite-based PFT map to LPJmL PFTs according to Köppen-Geiger climate zones.

Biomes are tropical (Tr = A, BSh, BWh climates), boreal (Bo = Dfc, Dfd, Ds, Dw, EF, ET), and temperate (Te = Cf, Cw, Cs, BSk, BWk, Dfa, Dfb). For example, the TrBE PFT was aggregated from the coverages of TreeBE and ShrubBE in the Tropical climate. For some PFTs, specific modifications were done (last column with explanation at the bottom).

|  | PFTs in the ESA CCI land cover-based maps ^11^ | | | | | | | | |  | |
| --- | --- | --- | --- | --- | --- | --- | --- | --- | --- | --- | --- |
| PFTs in LPJmL | Tree BE | Tree BD | Tree NE | Tree ND | Shrub BE | Shrub BD | Shrub NE | Shrub ND | Nat. grass | | Modif-ication |
| TrBE | Tr |  |  |  | Tr |  |  |  |  | | C |
| TrBR |  | Tr |  |  |  | Tr |  |  |  | | B |
| TeBE | Te \| Bo |  |  |  | Te \| Bo |  |  |  |  | | C |
| TeBS |  | Te |  |  |  | Te |  |  |  | | A, B |
| TeNE |  |  | Te \| Tr |  |  |  | Te \| Tr |  |  | | D |
| BoBS |  | Bo |  |  |  | Bo |  |  |  | | A |
| BoNE |  |  | Bo |  |  |  | Bo |  |  | | D |
| BoNS |  |  |  | Bo \| Te |  |  |  | Bo \| Te |  | |  |
| TrH |  |  |  |  |  |  |  |  | Tr | |  |
| TeH |  |  |  |  |  |  |  |  | Te | |  |
| PoH |  |  |  |  |  |  |  |  | Bo | |  |
| **Modifications:** For some PFTs, specific re-assignments of PFT fractions were done based on latitudes:  Boreal latitudes: BoLat = latitude > 53°N  Tropical latitudes: TrLat = latitude > 20°S and latitude < 17°N  Temperate latitudes: TeLat = remaining latitudes   1. TeBS to BoBS: Fractions > 0.3 of initially assigned TeBS in the BoLat were assigned to BoBS. 2. TeBS to TrBR: All fractions of initially assigned TeBS in the TrLat were assigned to TrBR. 3. TeBE to TrBE: All fractions of initially assigned TeBE in the TrLat were assigned to TrBE. 4. TeNE to BoNE: Fractions > 0.1 of initially assigned TeNE in the BoLat were assigned to BoNE. | | | | | | | | | | | |


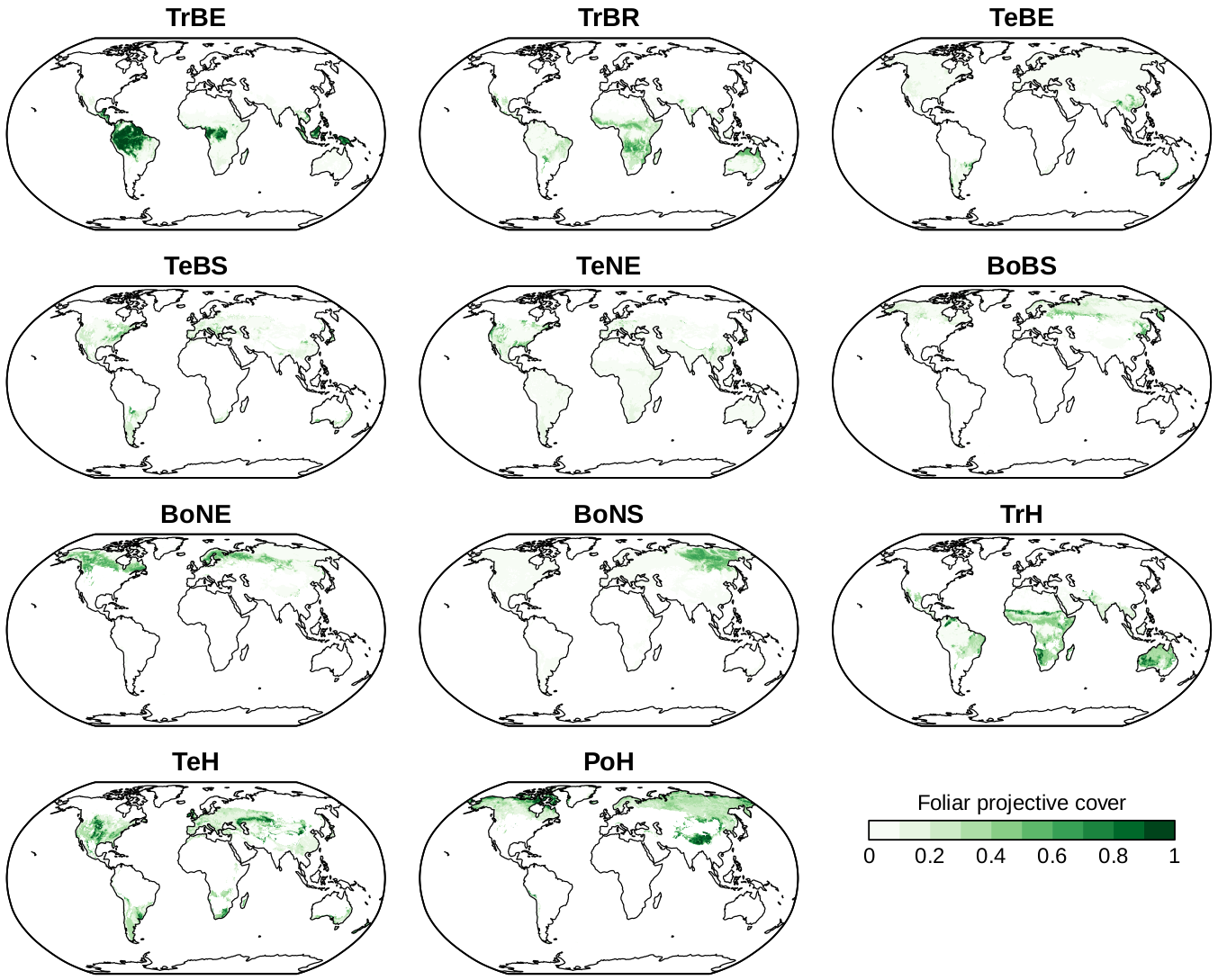


**Supplementary Figure 12:** Maps of PFTs according to the definition of LPJmL derived from the ESA CCI land cover-based PFT map.

# SI 5: Sampling of grid cells for the optimization

The sampling of grid cells that were used for the optimization was done for the PFTs of the boreal, temperate, and tropical zones, respectively. Grid cells were sampled for each zone. The sampling was done randomly but stratified by (1) dominant PFTs, (2) by the distribution of above-ground biomass of trees, and (3) by the level of vegetation dynamics. The procedure was as follows:

1. We derived statistics on PFTs for each grid cell from the satellite-based PFT maps (**Supplementary Figure 12**). Specifically, we computed the total fractional coverage of all PFTs (fVeg), the number of PFTs with > 10% coverage (NPFT, coverage > (fVeg * 0.1)), the PFT with maximum coverage (MaxPFT), and if the PFT with maximum coverage has dominance, i.e. coverage > (fVeg * 0.5) (HasDom).
2. We used the burned area map to select grid cells that burned in the earlier years of the optimization period to potentially constrain post-fire vegetation dynamics. Therefore we computed the cumulated burned area over 2001-2008 and assigned grid cells the flag “LargeFire” if the cumulated fractional burned area was > 0.4.
3. We then created a map with potential levels of vegetation dynamics (0 = no vegetation dynamics to 3 = high level of vegetation dynamics) (**Supplementary Figure 13**). The levels are based on the number of criteria that are fulfilled:
   1. Do more than two PFT exist (NPFT > 2)?
   2. Is there no dominant PFT (HasDom = FALSE)?
   3. Did large fires occur in 2001-2008 (LargeFire = TRUE)?

We then sampled 102 grid cells for each zone whereby 70 % of the grid cells were sampled per PFT and stratified by above-ground biomass (Sample 1), and 30 % were sampled for the different levels of vegetation dynamics (10 % for each level 1 to 3) (Sample 2).

1. Sample 1: For each PFT within a zone:
   1. We selected the grid cells where the PFT has maximum coverage (MaxPFT).
   2. With this PFT mask, we then masked the above-ground biomass map and computed the percentiles 0% (minimum), 20%, 40%, 60%, 80%, 100% (maximum) of biomass. This results in five quantile classes and represents the statistical distribution of tree biomass for a PFT.
   3. For each quantile class, we randomly sampled grid cells.
2. Sample 2: Within a climate zone we selected the grid cells for vegetation dynamics levels 1, 2 or 3 and randomly sampled grid cells.

This sampling scheme ensures that each PFT is included with its full statistical distribution of biomass and that also grid cells are included with potential competition between PFTs or post-fire vegetation dynamics.


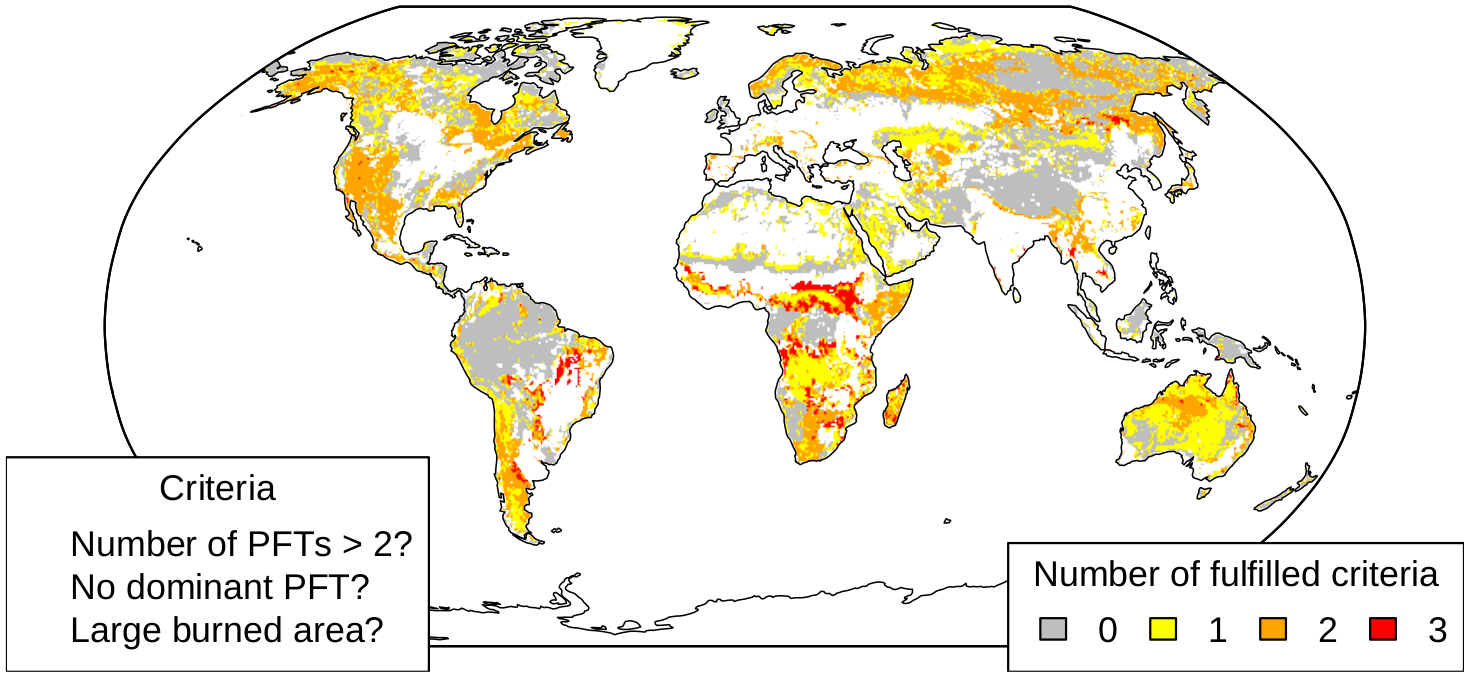


**Supplementary Figure 13:** Map to stratify the sampling of grid cells according to the level of vegetation dynamics.

White areas are either un-vegetated or had more than 20 % cropland cover.

# References

1. Forkel, M. *et al.* Identifying environmental controls on vegetation greenness phenology through model–data integration. *Biogeosciences* **11**, 7025–7050 (2014).

2. Schaphoff, S. *et al.* LPJmL4 – a dynamic global vegetation model with managed land – Part 1: Model description. *Geosci Model Dev* **11**, 1343–1375 (2018).

3. MacBean, N. *et al.* Strong constraint on modelled global carbon uptake using solar-induced chlorophyll fluorescence data. *Sci. Rep.* **8**, 1973 (2018).

4. Poulter, B. *et al.* Plant functional type mapping for earth system models. *Geosci Model Dev* **4**, 993–1010 (2011).

5. Thurner, M. *et al.* Large-scale variation in boreal and temperate forest carbon turnover rate related to climate. *Geophys. Res. Lett.* **43**, 4576–4585 (2016).

6. Thurner, M. *et al.* Evaluation of climate-related carbon turnover processes in global vegetation models for boreal and temperate forests. *Glob. Change Biol.* **23**, 3076–3091 (2017).

7. Thurner, M. *et al.* Carbon stock and density of northern boreal and temperate forests. *Glob. Ecol. Biogeogr.* **23**, 297–310 (2014).

8. Myneni, R. B., Knyazikhin, Y. & Park, T. MOD15A2 MODIS/Terra Leaf Area Index/FPAR 8-Day L4 Global 1km SIN Grid, Boston University and MODAPS SIPS, NASA. (2015).

9. Köhler, P., Guanter, L. & Joiner, J. A linear method for the retrieval of sun-induced chlorophyll fluorescence from GOME-2 and SCIAMACHY data. *Atmospheric Meas. Tech.* **8**, 2589–2608 (2015).

10. Avitabile, V. *et al.* An integrated pan‐tropical biomass map using multiple reference datasets. *Glob. Change Biol.* **22**, 1406–1420 (2016).

11. Li, W. *et al.* Gross and net land cover changes in the main plant functional types derived from the annual ESA CCI land cover maps (1992–2015). *Earth Syst. Sci. Data* **10**, 219–234 (2018).

12. Chuvieco, E. *et al.* Generation and analysis of a new global burned area product based on MODIS 250 m reflectance bands and thermal anomalies. *Earth Syst. Sci. Data* **10**, 2015–2031 (2018).

13. Tramontana, G. *et al.* Predicting carbon dioxide and energy fluxes across global FLUXNET sites with regression algorithms. *Biogeosciences* **13**, 4291–4313 (2016).
